# Comparative Metabolomics of *Mycoplasma bovis* and *Mycoplasma gallisepticum* Reveals Fundamental Differences in Active Metabolic Pathways and Suggests Novel Gene Annotations

**DOI:** 10.1128/mSystems.00055-17

**Published:** 2017-10-10

**Authors:** Y. Masukagami, D. P. De Souza, S. Dayalan, C. Bowen, S. O’Callaghan, K. Kouremenos, B. Nijagal, D. Tull, K. A. Tivendale, P. F. Markham, M. J. McConville, G. F. Browning, F. M. Sansom

**Affiliations:** a Asia-Pacific Centre for Animal Health, Melbourne Veterinary School, Faculty of Veterinary and Agricultural Sciences, The University of Melbourne, Parkville, VIC, Australia; b Metabolomics Australia, The Bio21 Molecular Science and Biotechnology Institute, The University of Melbourne, Parkville, VIC, Australia; University of California, San Diego

**Keywords:** carbon metabolism, genome annotation, metabolomics, mycoplasma

## Abstract

Mycoplasmas are simple, but successful parasites that have the smallest genome of any free-living cell and are thought to have a highly streamlined cellular metabolism. Here, we have undertaken a detailed metabolomic analysis of two species, *Mycoplasma bovis* and *Mycoplasma gallisepticum*, which cause economically important diseases in cattle and poultry, respectively. Untargeted gas chromatography-mass spectrometry and liquid chromatography-mass spectrometry analyses of mycoplasma metabolite extracts revealed significant differences in the steady-state levels of many metabolites in central carbon metabolism, while ^13^C stable isotope labeling studies revealed marked differences in carbon source utilization. These data were mapped onto *in silico* metabolic networks predicted from genome wide annotations. The analyses elucidated distinct differences, including a clear difference in glucose utilization, with a marked decrease in glucose uptake and glycolysis in *M. bovis* compared to *M. gallisepticum*, which may reflect differing host nutrient availabilities. The ^13^C-labeling patterns also revealed several functional metabolic pathways that were previously unannotated in these species, allowing us to assign putative enzyme functions to the products of a number of genes of unknown function, especially in *M. bovis*. This study demonstrates the considerable potential of metabolomic analyses to assist in characterizing significant differences in the metabolism of different bacterial species and in improving genome annotation.

**IMPORTANCE** Mycoplasmas are pathogenic bacteria that cause serious chronic infections in production animals, resulting in considerable losses worldwide, as well as causing disease in humans. These bacteria have extremely reduced genomes and are thought to have limited metabolic flexibility, even though they are highly successful persistent parasites in a diverse number of species. The extent to which different *Mycoplasma* species are capable of catabolizing host carbon sources and nutrients, or synthesizing essential metabolites, remains poorly defined. We have used advanced metabolomic techniques to identify metabolic pathways that are active in two species of *Mycoplasma* that infect distinct hosts (poultry and cattle). We show that these species exhibit marked differences in metabolite steady-state levels and carbon source utilization. This information has been used to functionally characterize previously unknown genes in the genomes of these pathogens. These species-specific differences are likely to reflect important differences in host nutrient levels and pathogenic mechanisms.

## Abstract (precis)

Mycoplasmas are pathogenic bacteria that cause serious chronic infections in production animals, resulting in considerable losses worldwide, as well as causing disease in humans. These bacteria have extremely reduced genomes and are thought to have limited metabolic flexibility, even though they are highly successful persistent parasites in a diverse number of species. The extent to which different *Mycoplasma* species are capable of catabolizing host carbon sources and nutrients, or synthesizing essential metabolites, remains poorly defined. We have used advanced metabolomic techniques to identify metabolic pathways that are active in two species of *Mycoplasma* that infect distinct hosts (poultry and cattle). We show that these species exhibit marked differences in metabolite steady-state levels and carbon source utilization. This information has been used to functionally characterize previously unknown genes in the genomes of these pathogens. These species-specific differences are likely to reflect important differences in host nutrient levels and pathogenic mechanisms.

## INTRODUCTION

*Mycoplasma* species (class *Mollicutes*) are simple bacteria that have massively reduced genomes and are thought to be among the simplest self-replicating organisms. They are typically extracellular parasites of their eukaryotic hosts, causing persistent infections and chronic disease (1). A number of recent studies have suggested that the reduction in genome complexity in mycoplasmas is associated with a concomitant reduction in their metabolic capacity (2–4), resulting in an increased dependency on their host for many complex nutrients. Nonetheless, mycoplasmas can inhabit a wide variety of different hosts, suggesting that they have adapted to different nutrient environments. *Mycoplasma gallisepticum* and *Mycoplasma bovis* infect two distinct hosts—poultry and ruminants—but both cause significant disease and are a major cause of economic loss worldwide. *M. gallisepticum* is an important pathogen of chickens and turkeys, causing chronic respiratory disease (CRD) (5), and it is also an emerging cause of disease in some wild birds (6). *M. bovis* is a major cause of chronic calf pneumonia (7, 8), contributes to complex pneumonia in older cattle (7, 9), and is also an important cause of mastitis, arthritis, otitis media, keratoconjunctivitis, meningitis, abortion, and infertility (9, 10). Usually thought of as mucosal pathogens, there is some evidence that some mycoplasmas can invade host cells (11, 12). How, and to what extent, different *Mycoplasma* species adapt metabolically to these different host niches remains poorly defined.

While genome-wide metabolic reconstructions of different *Mycoplasma* species support the notion that these bacteria have a greatly reduced metabolic capacity, many genes in these bacteria remain uncharacterized, and direct analyses of the metabolome of this class of bacteria have been limited. Metabolomic analyses are particularly useful for identifying novel or unanticipated metabolic reactions/pathways, as well as for evaluating the extent to which these bacteria can utilize exogenous metabolites and regulate metabolic pathways under different growth conditions. In this study, we aimed to identify differences in the metabolic pathways of two species of *Mycoplasma* that infect distinct hosts (poultry and cattle) and to use this information to assign likely functions to previously unknown genes in the genomes of these pathogens. We performed a comparative, multiplatform analysis of the steady-state metabolomes of *M. gallisepticum* and *M. bovis* and integrated these data with ^13^C stable isotope labeling experiments using multiple labels (glucose, glycerol, pyruvate) to elucidate specific functional metabolic pathways and identify preferential use of different carbon sources by each species. These data were used to identify previously unannotated enzymes, including multifunctional enzymes as well as transporters, and to highlight significant differences in the metabolism of these pathogenic species that may underpin their tropism for different hosts.

## RESULTS

### Identification of metabolites in *M. bovis* and *M. gallisepticum*.

**FIG 1:**
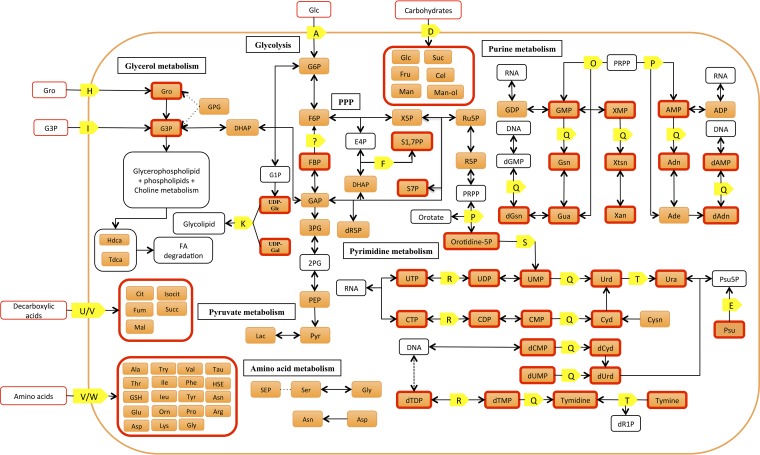
Condensed metabolic map of *M. bovis* constructed in this study (for the full metabolic map, see Fig. S3). Orange boxes indicate metabolites detected in this study (a red outline indicates the metabolite supports novel enzyme annotation). Yellow boxes indicate novel enzyme annotations arising from this work (for a complete list of new annotations, see Table S2 and Fig. S1). Annotations: A, phosphotransferase glucose transporter; D, CUT-2; E, PFK family carbohydrate kinase/pseudouridine kinase; F, fructose bisphosphate aldolase; H, glycerol ABC transporter; I, *sn*-glycerol-3-phosphate transport system; K, glycosyl transferase; O, hypoxanthine phosphoribosyltransferase; P, adenine/orotate phosphoribosyltransferase; Q, 5′-nucleotidase/nicotinamide ribonucleotide phosphohydrolase; (R) pyruvate kinase; (S) orotidine 5′-phosphate decarboxylase; T, pyrimidine phosphorylase; U, malate transporter; V, DAACS family transporter; W, LAT family transporter. The metabolite abbreviations are listed in Table S4.

**FIG 2:**
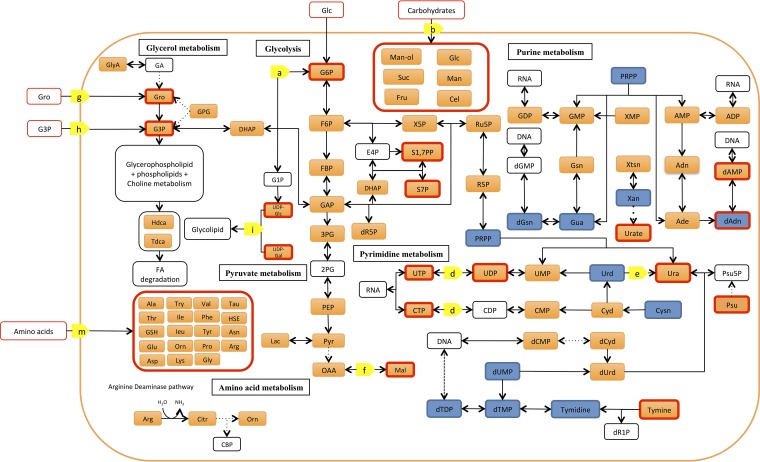
Condensed metabolic map of *M. gallisepticum* (updated from the map inferred by Vanyushkina et al. [4]; for the full metabolic map see Fig. S4). Orange boxes indicate metabolites detected in this study (a red outline indicates that the metabolite supports novel enzyme annotation). Blue boxes indicate metabolites previously detected (4). Yellow boxes indicate novel enzyme annotations arising from this work (for a complete list of new annotations, see Table S3 and Fig. S2). Annotations: a, phosphoglucomutase; b, ribose ABC transporter; d, nucleoside diphosphate kinase; e, uridine phosphorylase; f, malate dehydrogenase; g, glycerol uptake facilitator protein; h, *sn*-glycerol-3-phosphate ABC transport system; i, glycosyl transferase; m, amino acid permease. Metabolite abbreviations are listed in Table S4.

*M. bovis* and *M. gallisepticum* were grown axenically under identical culture conditions and harvested while in logarithmic growth. Bacterial metabolism was rapidly quenched by immersion of an aliquot of the culture in a dry ice-ethanol bath and transfer to an ice slurry, minimizing cell lysis and metabolite leakage that can occur when using other metabolite quenching techniques (4). Analysis of polar metabolite extracts by gas chromatography-mass spectrometry (GC/MS) and liquid chromatogrpahy-MS (LC/MS) resulted in the identification of a total of 132 metabolites (see Table S1 in the supplemental material). The majority of these metabolites (84/111 from *M. bovis* and 89/110 from *M. gallisepticum*) were identified based on retention time and mass spectra compared to authentic standards (Table S1). As expected, these analytical platforms provided overlapping and complementary coverage of the bacterial metabolomes. The majority of the metabolites identified in *M. bovis* (92/105) and *M. gallisepticum* (94/104) were annotated onto metabolic maps for these species (Fig. 1 and 2), demonstrating good coverage of several pathways in central carbon metabolism, including glycolysis, the pentose phosphate pathway (PPP), purine and pyrimidine metabolism, glycerol metabolism, and amino acid metabolism. Interestingly, a number of metabolites were detected that were not predicted from the genome annotation. Specifically, a number of organic acids linked to the tricarboxylic acid (TCA) cycle, including fumarate, malate, and succinate (*M. bovis* only) were detected by one or both analytical platforms. These metabolites have previously been detected in *M. pneumoniae* (2), but none of the mycoplasma genomes contain enzymes for a TCA cycle, suggesting that they are likely to have been scavenged from the medium. Other metabolites detected in these analyses, for which there are no predicted enzymes in the genome, include glucuronic acid, glucosamine and glucosamine-1-phosphate, galactonofuranose-6-phosphate, *myo*-inositol, and some nonphosphorylated sugars, such as fructose. These may be present in the mycoplasma cell simply due to uptake from the medium (for example, we detected glucuronic acid, *myo*-inositol, and fructose in our medium analyses [Table S1]) or potentially be produced by currently unknown pathways.

10.1128/mSystems.00055-17.5 TABLE S1  Metabolites identified in *M. gallisepticum* and *M. bovis* and MB medium analyses. Download TABLE S1, PDF file, 0.1 MB. Copyright © 2017 Masukagami et al. 2017 Masukagami et al. This content is distributed under the terms of the Creative Commons Attribution 4.0 International license.

10.1128/mSystems.00055-17.1 FIG S1  Multiple sequence alignments of proteins (see Table S2) that were assigned new enzyme annotations in this study for *M. bovis*. Multiple sequence alignments were constructed for the assigned proteins in *M. bovis* with homologous proteins identified in other mycoplasma and bacterial species by using Clustal Omega (http://www.ebi.ac.uk/Tools/msa/clustalo/). Numbers on the right indicate the position of the adjacent amino acid residue. An asterisk indicates an amino acid that is conserved in all aligned sequences, a colon indicates a substitution with a very similar amino acid, and a full stop (a period) indicates at least one substitution with a similar amino acid. Dashed lines indicate gaps in the amino acid sequence alignment. The active site for each enzyme is highlighted in red. Download FIG S1, PDF file, 0.1 MB. Copyright © 2017 Masukagami et al. 2017 Masukagami et al. This content is distributed under the terms of the Creative Commons Attribution 4.0 International license.

10.1128/mSystems.00055-17.6 TABLE S2  Novel putative enzymes annotated onto the *M. bovis* metabolic map in this study. Download TABLE S2, PDF file, 0.1 MB. Copyright © 2017 Masukagami et al. 2017 Masukagami et al. This content is distributed under the terms of the Creative Commons Attribution 4.0 International license.

10.1128/mSystems.00055-17.7 TABLE S3  Novel putative enzymes annotated onto the *M. gallisepticum* metabolic map in this study (the references for Tables S2 and S3 are listed). Download TABLE S3, PDF file, 0.2 MB. Copyright © 2017 Masukagami et al. 2017 Masukagami et al. This content is distributed under the terms of the Creative Commons Attribution 4.0 International license.

10.1128/mSystems.00055-17.2 FIG S2  Multiple sequence alignments of proteins (see Table S3) that were assigned new enzyme annotations in this study for *M. gallisepticum*. Multiple sequence alignments were constructed for the assigned proteins in *M. gallisepticum* with homologous proteins identified in other mycoplasma and bacterial species by using Clustal Omega (http://www.ebi.ac.uk/Tools/msa/clustalo/). Numbers on the right indicate the position of the adjacent amino acid residue. An asterisk indicates an amino acid that is conserved in all aligned sequences, a colon indicates a substitution with a very similar amino acid, and a full stop (a period) indicates at least one substitution with a similar amino acid. Dashed lines indicate gaps in the amino acid sequence alignment. Download FIG S2, PDF file, 0.1 MB. Copyright © 2017 Masukagami et al. 2017 Masukagami et al. This content is distributed under the terms of the Creative Commons Attribution 4.0 International license.

10.1128/mSystems.00055-17.3 FIG S3  Complete metabolic map of *M. bovis* that was constructed in this study. Orange boxes indicate detected metabolites. Yellow boxes indicate novel annotations from this study. Green boxes indicate previous annotations in the KEGG database (39). Download FIG S3, PDF file, 1.2 MB. Copyright © 2017 Masukagami et al. 2017 Masukagami et al. This content is distributed under the terms of the Creative Commons Attribution 4.0 International license.

10.1128/mSystems.00055-17.4 FIG S4  Complete metabolic map of *M. gallisepticum* that was constructed in this study (updated from the map inferred by Vanyushkina et al. [4]). Orange boxes indicate detected metabolites. Pink boxes indicate previously detected metabolites (4). Yellow boxes indicate novel annotations from this study. Green boxes indicate previous annotations in the KEGG database (39). Download FIG S4, PDF file, 1.1 MB. Copyright © 2017 Masukagami et al. 2017 Masukagami et al. This content is distributed under the terms of the Creative Commons Attribution 4.0 International license.

10.1128/mSystems.00055-17.8 TABLE S4  Metabolite abbreviations used in Fig. 1, 2, and 3 in the text. Download TABLE S4, PDF file, 0.1 MB. Copyright © 2017 Masukagami et al. 2017 Masukagami et al. This content is distributed under the terms of the Creative Commons Attribution 4.0 International license.

### Metabolite differences between *M. bovis* and *M. gallisepticum.*

**FIG 3:**
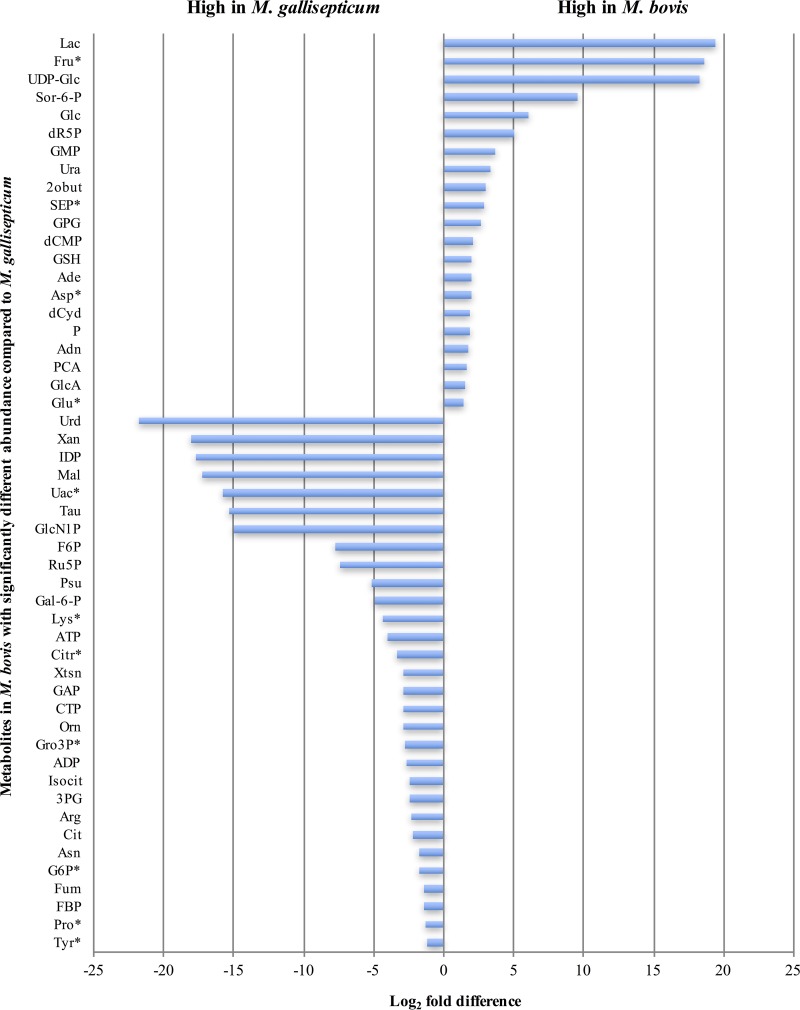
Metabolites differing significantly in abundance between *M. bovis* and *M. gallisepticum* (*P* < 0.05, BH-adjusted unpaired *t* test). Metabolites with asterisks were validated as significantly different in both the GC/MS and LC/MS analyses. Metabolite abbreviations are listed in Table S4.

Significant differences (*P* < 0.05, Benjamini-Hochberg [BH]-corrected *t* test) were observed in the metabolite profiles of the two *Mycoplasma* species, with 42% and 39% of metabolites detected in the GC/MS and LC/MS analyses differing by more than log_2_ 1.2-fold (30.5% of the total metabolites detected across both platforms) (Fig. 3). In particular, intermediates in glycolysis, the putative TCA cycle, and the arginine deiminase (ADI) pathway (including arginine, citrulline, and ornithine) were elevated in *M. gallisepticum* compared to *M. bovis*. In contrast, free sugars, such as glucose, fructose, mannose, and sucrose, were found at higher abundances in *M. bovis*. The accumulation of these sugars may reflect an increased transport capacity for neutral sugars/disaccharides in *M. bovis*, decreased conversion of these sugars to their phosphorylated forms, and/or elevated expression of phosphatases that act on sugar phosphate pools. The presence of intermediates in the ADI pathway in *M. gallisepticum* was also surprising, as this species is not predicted to contain a complete ADI pathway (3, 4).

Significant differences were found in the levels of major nucleobases, nucleosides, and nucleotides. In particular, AMP, ADP, and ATP were found at higher abundances in *M. gallisepticum*, while adenine, adenosine, and the nucleotides GMP, CMP, UMP, dCMP, and dUMP were all elevated in *M. bovis*. Uric acid and pseudouridine, metabolites that are unique to DNA/RNA metabolism, were only detected in *M. gallisepticum*.

Metabolites such as lactate and 2-oxobutanoate, which are generated during pyruvate and propanoate metabolism, were detected in higher abundances in *M. bovis*. Abundances of fatty acids, including palmitic acid, myristic acid, and glycerophosphoryl glycerol (GPG), which is the phospholipid component of the mycoplasma membrane, were significantly higher in *M. bovis*. UDP-glucose, which has previously been shown in *M. gallinarum* to act as a donor for sterol glucoside synthesis (13), was observed at higher levels in *M. bovis*. Intermediates of amino acid metabolism, including *O*-phospho-l-serine (SEP) and pyroglutamate (PCA), were significantly higher in *M. bovis*.

### Both species of mycoplasma utilize [^13^C]glucose and [^13^C]glycerol.

**FIG 4:**
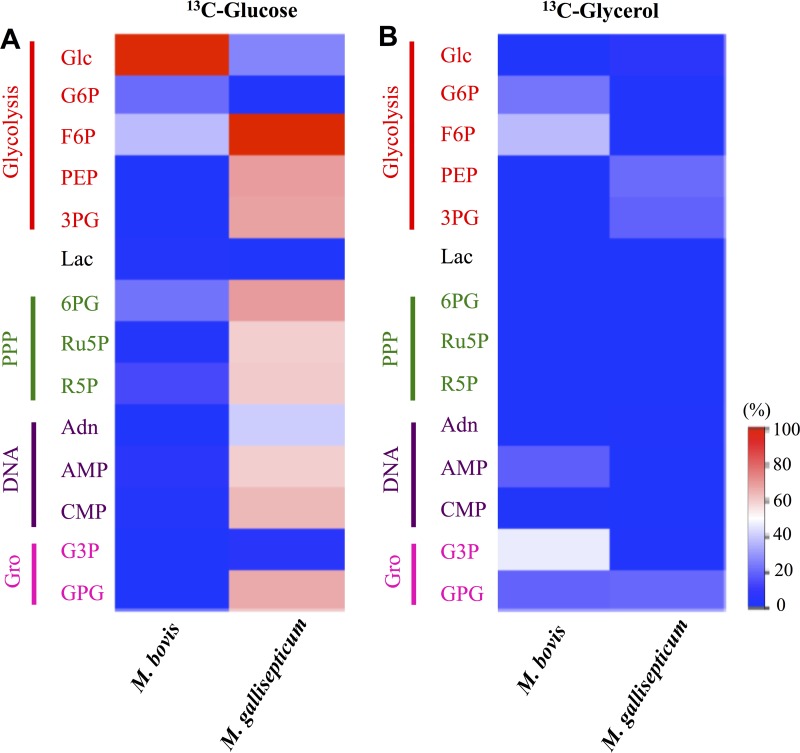
Metabolites labeled in *M. bovis* and *M. gallisepticum* after culture with [^13^C]glucose (A) or [^13^C]glycerol (B). The scale indicates the proportion (percentage) of metabolite containing the label. Values were normalized to the ^13^C/^12^C ratio in the medium. Abbreviations: Glc, glucose; G6P, glucose 6-phosphate; F6P, fructose 6-phosphate; PEP, phosphoenolpyruvate; 3PG, 3-phosphoglycerate; Lac, lactate; 6PG, 6-phosphogluconate; Ru5P, ribulose 5-phosphate; R5P, ribose 5-phosphate; Adn, adenosine; G3P, glycerol 3-phosphate; GPG, glycerophosphoryl glycerol.

To define the extent to which different metabolites identified in mycoplasma extracts were synthesized *de novo* or scavenged from the medium, both mycoplasma species were metabolically labeled with [^13^C]glucose, and ^13^C enrichment in intracellular metabolites was determined by GC/MS. Intriguingly, [^13^C]glucose was effectively taken up by *M. bovis*, as shown by efficient steady-state labeling of the intracellular glucose pool (78%). However, only low levels of label were detected in downstream glycolytic and pentose phosphate pathway intermediates (Fig. 4A), indicating that this species may preferentially utilize other carbon sources for energy generation, resulting in cross-membrane exchange of intracellular and extracellular glucose pools. In marked contrast, [^13^C]glucose was rapidly taken up by *M. gallisepticum* and shunted into intermediates in glycolysis, the PPP, and DNA/RNA metabolism (Fig. 4A). Incorporation of label into lactate and malate was not detected, which suggests that the multifunctional lactate/malate dehydrogenase (LDH/MDH) (MGA_0746) is inactive under these growth conditions. The label was also not incorporated into the organic acids fumarate and citrate, confirming the absence of a functional TCA cycle in these bacteria. These intermediates may be scavenged from the culture medium, although their function in cellular metabolism remains undefined. The strong labeling (>50%) of PPP intermediates, as well as detection of M + 3 and M + 5 ions for adenosine and AMP, is consistent with an active purine salvage pathway in this species and the absence of *de novo* synthesis (14).

To investigate whether *M. bovis* uses other carbon sources, both species were cultivated in the presence of [^13^C]glycerol. Significant uptake of [^13^C]glycerol and incorporation into glycerol-3-phosphate (G3P) and hexose phosphates were observed in *M. bovis*, suggesting that this species may use nonhexose carbon sources and gluconeogenesis under these growth conditions (Fig. 4B). In contrast, [^13^C]glycerol labeling of *M. gallisepticum* did not lead to detectable labeling of hexose phosphates, although GPG and intermediates in lower glycolysis, such as phosphoenolpyruvate (PEP) and 3-phosphoglycerate (3PG), were partially labeled (Fig. 4B). No uptake or incorporation of label of another carbon source, [^13^C]pyruvate, was detected in either species (data not shown). Taken together, these data suggest that the fluxes in central carbon metabolism differ markedly in these two *Mycoplasma* species.

### Differential depletion and secretion of metabolites by *M. bovis* and *M. gallisepticum.*

**FIG 5:**
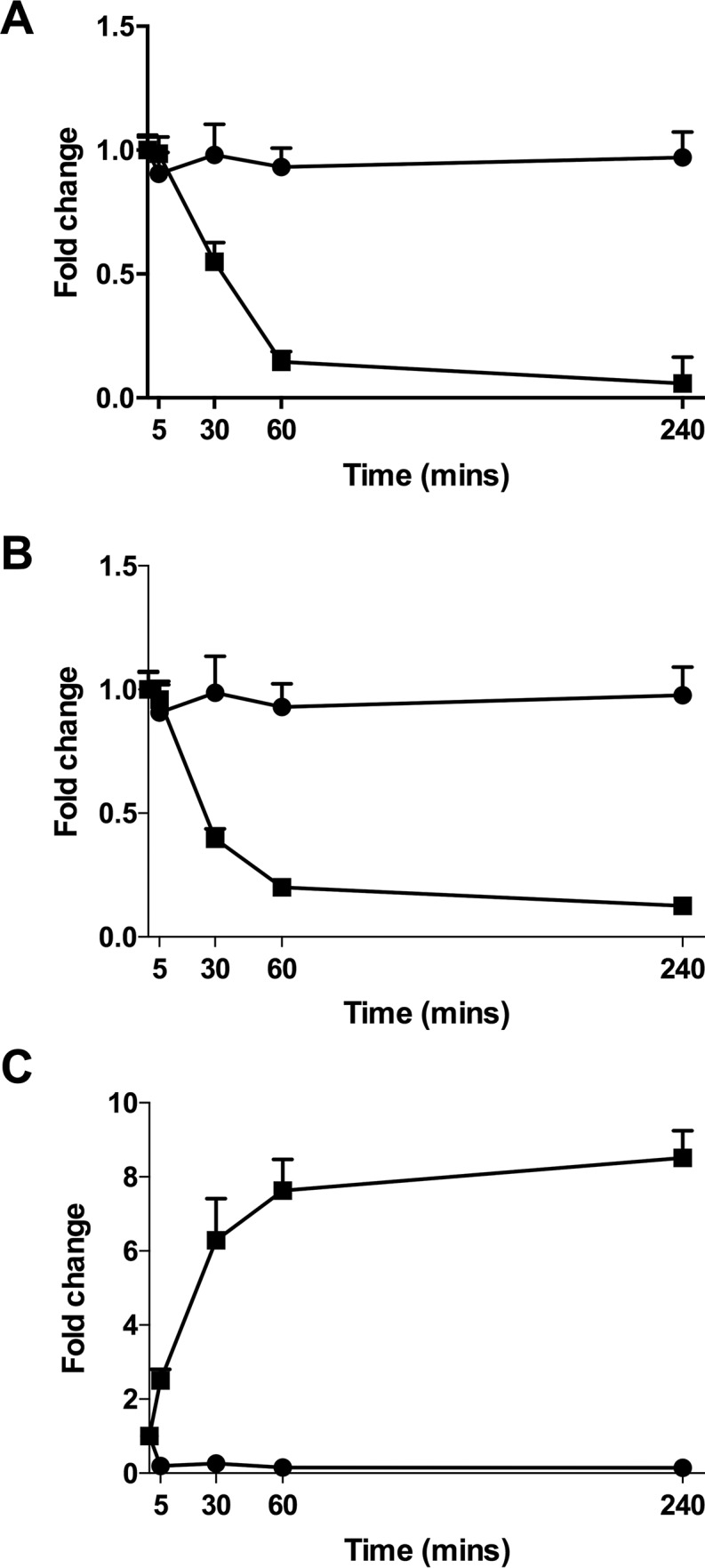
Level of glucose (A), fructose (B), and lactate (C) in medium from *M. gallisepticum* (squares) and *M. bovis* (circles) cultures (relative to the average abundance at *t* = 0). Culture supernatants were analyzed by GC-MS at selected time points up to 4 h after resuspension of late-logarithmic-phase bacteria in fresh medium (*n* ≥ 3 at each time point; error bars indicate standard deviations).

In order to further elucidate differences in carbon source utilization between the two species, we resuspended late-logarithmic-phase bacteria in fresh culture medium and measured changes in metabolite levels in the medium over 4 h (Fig. 5). Strikingly, *M. gallisepticum* rapidly depleted the culture medium of both fructose and glucose, whereas lactate levels rose by more than 5-fold after 30 min, suggesting rapid sugar fermentation and secretion of lactate as a waste product. In contrast, minimal changes in the levels of either sugar were observed in the medium containing *M. bovis*, and instead available lactate was rapidly depleted, further suggesting a clear difference in utilization of carbon sources by the two species.

### Identification of new mycoplasma gene functions related to metabolism.

Our metabolomic analyses highlighted a number of unanticipated metabolic pathways and transporter systems from that inferred by previous genome-wide metabolic reconstructions (4). Detailed analysis of the *M. bovis* genome allowed us to ascribe enzyme functions to 23 genes and transporter functions to a further 6 genes that previously had no annotated function (Fig. 1; Table S2). Similarly, further annotation of the *M. gallisepticum* genome resulted in provisional annotation of 14 new enzymes and 3 transporters, compared to the annotation inferred by Vanyushkina et al. (4) (Fig. 2; Table S3).

The lack of appreciable labeling of glycolytic intermediates downstream of fructose-6-phosphate (F6P) in [^13^C]glucose-fed *M. bovis* suggested that this species may lack the first dedicated glycolytic enzyme, phosphofructokinase (PFK), and this would be consistent with genome annotations (15). In contrast, the labeling of hexose phosphates with [^13^C]glycerol (Fig. 4B) suggested that this species can shunt glycerol metabolites into gluconeogenesis. We were unable to identify a gene with homology to fructose 1,6-bisphosphatase (FBPase), despite a BLAST search of the *M. bovis* genome using amino acid sequences of type I, II, and III FBPases, although incorporation of ^13^C label from [^13^C]glycerol into F6P indicated this reaction was occurring.

We showed that *M. bovis* can utilize glycerol as a major carbon source and that this metabolite is catabolized in both the lower glycolytic and gluconeogenic pathways. We annotated a transporter gene operon encoding a putative *sn*-glycerol-3-phosphate ABC transporter, comprising the genes MBOVPG45_0307, MBOVPG45_0308, MBOVPG45_0309, and MBOVPG45_0310. In *M. bovis*, we identified genes encoding a phosphotransferase system (PTS) transporter system for glucose uptake, as well as a carbohydrate family ABC transporter (CUT-2) (Fig. 1; Table S2). Although we identified genes for both transporters in the *M. bovis* genome, the detection of large pools of unphosphorylated glucose in *M. bovis*, which is rapidly exchanged with [^13^C]glucose (Fig. 4A), indicated that these bacteria predominantly utilize the CUT-2 neutral hexose ABC transporter to acquire sugars.

Organic acids and amino acids were detected in our *M. bovis* metabolite analyses, and based on our bioinformatic analyses (Table S2), we assigned the DAACS family transporter (encoded by MBOVPG45_0568) and the LAT family ABC transporter (encoded by MBOVPG45_0533) as putative transporters of these substrates (Fig. 1). Malate was also detected and, based on orthology with a malate permease in *Mycoplasma fermentans* (MFE_01530), as well as with the product of the *Clostridium cadaveris* malate permease gene (coverage of 95%, E value of 2e−18, identity of 25%), we annotated MBOVPG45_0327 as a putative malate permease gene (it is currently annotated as an AEC family transporter gene) (Fig. 1; Table S2 and Fig. S1).

The *M. bovis* gene MBOVPG45_0796 is currently annotated as the adenine phosphoribosyltransferase gene (*apt*), but the predicted amino acid sequence contains the conserved phosphoribosyltransferase type 1 domain (Table S2). In other organisms, this domain is found in a diverse range of phosphoribosyl transferases and regulatory proteins of the nucleotide synthesis and salvage pathway, including orotate phosphoribosyltransferase. The detection of orotidine 5′-phosphate in the *M. bovis* metabolic profile suggests the protein may be multifunctional (and also have orotate phosphoribosyltransferase activity), connecting the PPP to pyrimidine metabolism (Fig. 1; Table S2).

The *ushA* gene (MBOVPG45_0690) was identified in *M. bovis* based on its predicted amino acid sequence similarity with the predicted protein sequence of the translated *Escherichia coli ushA* gene and also by domain analysis of the amino acid sequence (Table S2). The enzyme encoded by this gene would allow *M. bovis* to convert pyrimidine and purine monophosphates to pyrimidine and purine bases (Fig. 1). No genes encoding this enzyme are annotated within the genomes of *M. gallisepticum* and *M. pneumoniae*; also, we could not detect them in our bioinformatic analysis.

## DISCUSSION

We have used a combination of genomic and metabolomic analyses to further investigate the metabolic potential of mycoplasmas and identify potential species-specific differences in two pathogenic species with distinct host ranges. Assignation of the putative enzymes using metabolic mapping has been described previously for the human pathogen *Mycoplasma pneumoniae* (2, 3) and for three different species within the *Mollicutes*, including *M. gallisepticum* (4), although the previous analysis of *M. gallisepticum* was restricted to use of LC/MS. The parallel use of two analytical platforms (GC/MS and LC/MS), together with ^13^C stable isotope labeling experiments, allowed us to predict the presence of a number of key metabolic functions and to identify putative gene candidates in the respective genomes of the two mycoplasma species. By linking the metabolites identified in this study with genomic analyses of each species and the existing literature, we reconstructed the metabolic pathways of *M. bovis* by assigning putative enzyme activities and added newly annotated enzymes onto the pathways previously published for *M. gallisepticum* (4).

### Differential uptake and utilization of carbon sources in *M. bovis* and* M. gallisepticum.*

Our comparative metabolite profiling and [^13^C]glucose-labeling studies with *M. gallisepticum* and *M. bovis* highlighted clear differences in the way each of these bacteria catabolizes glucose (14, 16). Specifically, *M. gallisepticum* actively takes up exogenous glucose and accumulates large intracellular pools of hexose phosphate, indicating operation of the PTS glucose and carbohydrate transporter systems (17). These sugar phosphates are subsequently catabolized in the glycolytic and PPP, generating ATP and reducing equivalents, respectively. In contrast, *M. bovis* appears to primarily generate phosphorylated sugars via gluconeogenesis, even when exogenous glucose levels are high. These bacteria can take up glucose and other neutral sugars (Table S1; Fig. 4), possibly via the nonspecific carbohydrate ABC transporter CUT-2 (Table S2). Somewhat unexpectedly, steady-state analyses demonstrated glucose and fructose levels were higher in *M. bovis* cells than in *M. gallisepticum* cells, even though medium analyses found that these sugars were depleted much more rapidly from the medium by *M. gallisepticum*. However, *M. bovis* lacks a glucokinase homologue, providing an explanation for the low rate of conversion of exogenous glucose to G6P and other sugar phosphates and the absence of continuous uptake. Interestingly some of the internalized glucose is nonetheless converted to G6P, indicating operation of a PTS in this species and/or a cryptic gene encoding hexose/glucokinase activity. The sugar phosphates generated via gluconeogenesis or low-level PTS/kinase activities in *M. bovis* are primarily catabolized in the PPP, producing intermediates such as ribose-5-phosphate (R5P; 10%) and nucleotides for DNA and RNA synthesis. The absence of any flux into the glycolytic pathways is consistent with the absence of a PFK homologue in this species. Although we observed experimental evidence for FBPase activity, we could not identify a candidate gene in the *M. bovis* genome. *Archaea* and some primitive bacteria utilize a dual-function enzyme, FBP aldolase/phosphatase, for this reaction (18), and it is possible a similar situation occurs in *M. bovis*. It is notable that neither *M. gallisepticum* nor *M. bovis* contains a recognizable gene for G6PDH, the first enzyme in the oxidative PPP, and previous studies have suggested that mycoplasmas may lack a functional oxidative PPP (15, 19, 20). However, our studies suggest that this pathway is active in both species, although the origins of the hexose phosphates that drive this pathway may differ significantly.

The genomes of all mycoplasmas lack genes encoding a canonical TCA cycle, although it has been suggested that *M. gallisepticum* has a multifunctional lactate and malate dehydrogenase (LDH/MDH) (MGA_0746) (14, 21, 22) (Table S3). The LDH/MDH may have a role in regenerating the cellular levels of NAD^+^ that are required to sustain glycolysis, a possibility supported by our finding that *M. gallisepticum* contains elevated levels of malate (Fig. 3). Paradoxically, malate was not labeled in [^13^C]glucose-fed *M. gallisepticum*, which could indicate that this enzyme is not active *in vitro*, or that rapid exchange with unlabeled extracellular pools of malate occurs. In that respect, we identified a putative malate permease (encoded by MBOVPG45_0327), which is annotated as an AEC family transporter in *M. bovis* and may be responsible for malate transport. More broadly, our [^13^C]glucose-labeling studies confirmed that glucose was not converted into intermediates of TCA metabolism in either species. Citrate, fumarate, and malate are present in the culture medium and appear to be imported by *M. bovis*, but the functional significance of this uptake is unknown. We were also able to annotate the protein encoded by MBOVPG45_0568 as a putative DAACS transporter, which is likely to be responsible for our observation of importation of amino acids and carboxylic acids into *M. bovis*.

The greater abundance of lactate in *M. bovis* and the rapid uptake from the medium suggest it may utilize lactate as a preferred carbon source, as suggested previously (15), while in *M. gallisepticum* production of lactate during pyruvate fermentation (generating protons) may cause the drop in pH seen during culture (14). Uptake of lactate by *M. bovis* may not only be for energy, but also for lactate oxidation, producing hydrogen peroxide (H_2_O_2_), which is thought to be a virulence factor in some mycoplasmas (23).

Our results suggest that *M. bovis* preferentially uses nonsugar carbon sources. This may reflect the biochemical environment in its ruminant host, in which volatile fatty acids and lactate (rather than glucose) are the main sources of energy absorbed from the gastrointestinal tract (16). In an attempt to identify the preferred nutrients of *M. bovis*, [^13^C]glycerol and [^13^C]pyruvate labeling studies were conducted, as these two nutrients have been suggested to be the preferred carbon sources for *M. bovis* (16). Detection of labeled G6P, F6P, and G3P in *M. bovis* demonstrated that glycerol was converted to G3P and DHAP via glycerol 3-phosphate dehydrogenase (encoded by MBOVPG45_0057) prior to channeling into gluconeogenesis. We also demonstrated the carbon backbones derived from [^13^C]glycerol can be used to sustain nucleotide biosynthesis via the salvage pathway. The extent of labeling of G3P and GPG in *M. bovis* suggests that glycerol is preferentially directed toward incorporation into phospholipid synthesis. In contrast, in *M. gallisepticum*, glycerol appears to be directed through glycolysis toward fermentation, demonstrated by the detection of labeled PEP and 3PG (16). A recent study demonstrated that *M. gallisepticum* could grow on glycerol-containing medium (that lacked glucose) and suggested that H_2_O_2_ production resulting from conversion of glycerol to DHAP may also play a role in virulence of this species (12). Interestingly, we did not detect any labeling in polar metabolites with [^13^C]pyruvate in either species (data not shown), similar to previous work in *M. agalactiae* strain PG2 (15), suggesting that pyruvate is not preferred over glucose (which was also present in the medium).

Detection of label from [^13^C]glycerol incorporated into GPG in both species suggests that both *Mycoplasma* species synthesize phospholipids, presumably for incorporation into the cell membrane (24), and future analysis of the lipid profile of mycoplasmas would be useful. Glucose may also be utilized for phospholipid synthesis through the earlier stage of glycerolipid metabolism, via DHAP to G3P, and then incorporated into GPG, rather than through conversion of G6P to G1P and incorporation into UDP-glucose and then glycolipid in *M. gallisepticum*. UDP-glucose, which is a substrate for glycolipid biosynthesis (25), was detected in greater abundance in *M. bovis*, and enzymes for utilization of UDP-hexose were annotated in our *M. bovis* analysis, suggesting that glucose is used for glycolipid synthesis in this organism. UDP-glucose could also be incorporated into capsular biosynthesis in *M. bovis*, as reported in *M. mycoides* subsp. *mycoides* (26).

The detection of 3-methyl-2-oxobutanoic acid in both species may be a result of degradation of amino acids. However, we were unable to identify a gene encoding an enzyme that would be able to generate this product. Similar breakdown products, *S*-ribosyl-l-homocysteine and 5-oxopentanoate, were also detected in a previous study (4). Other metabolites that have been detected but are also not predicted from genome analysis include glucosamine, GlcN-1-P, and Gal6-P, which indicate hexose/hexosamine kinase activities.

### Purine and pyrimidine metabolism.

Uric acid and pseudouridine were only detected in *M. gallisepticum*. Xanthine was also more abundant in *M. gallisepticum*. Uric acid is a derivative of purine metabolism generated by oxidization of xanthine; it is not as toxic as ammonia, but it does alkalinize the cytoplasm. *M. gallisepticum* cells presumably accumulate xanthine and produce uric acid to alkalinize their cytoplasm, allowing them to persist under the acidic conditions that normally prevail in their site of replication in their host (4). We could not identify the enzymes responsible for generation of pseudouridine and uric acid in our analysis of the *M. gallisepticum* genome, suggesting that there may be dual-function enzymes capable of catalyzing these reactions.

We identified a putative novel 5′-nucleotidase (encoded by MBOVPG45_0690) in *M. bovis* that would allow conversion of nucleotide monophosphates to nucleosides. The 5′-nucleotidase function has been assigned to thymidine kinase in *M. gallisepticum* (4), but this enzyme and function are absent in *M. pneumoniae* (2, 3). The relatively higher abundance of adenosine, deoxycytidine, and guanosine, derived from (d)NMPs, in *M. bovis* may reflect differing levels of 5′-nucleotidase activity in the two species, perhaps due to the differing enzymes. Our GC/MS analysis revealed incorporation of label from [^13^C]glucose in adenosine and AMP in an M + 3 and M + 5 ion-labeling pattern, demonstrating the two different streams of incorporation into ribose 5-phosphate, either from G6P or from GAP. This supports the conclusion that *M. gallisepticum* possesses a functional PPP that incorporates ribose phosphate into adenosine and AMP through the purine salvage pathway, as *Mollicutes* are unable to synthesize purines and pyrimidine *de novo* (14, 19, 27, 28). Furthermore, our annotation of the *apt* gene as the orotate phosphoribosyltransferase gene (*pyrE*) in the *M. bovis* pyrimidine metabolism pathway suggests that the orotate-related pathway is functional, in addition to the uracil phosphoribosyltransferase (*upp*)-dependent uracil-to-UMP conversion, although probably only when orotate and PRPP are available, as we could not annotate the other enzyme involved in *de novo* pyrimidine synthesis. The homologue of this gene in *Mycoplasma penetrans* was previously annotated as *pyrE* and thus considered to play a role in orotate-related metabolism (29).

### Conclusions.

We have characterized the phenotypic differences between two different pathogenic *Mycoplasma* species by integrating comprehensive metabolome data with genomic data. We constructed a metabolic map for *M. bovis* and refined the *M. gallisepticum* metabolic map described previously (4) by defining additional pathways and annotating novel putative enzymes, including assigning multifunctional enzymes, which are thought to be prevalent in mycoplasmas (3). Integration of metabolomic data with genetic and protein analyses provided robust information for the elucidation of the basic physiology of mycoplasmas, as it characterized the final products of the “–omics” cascade. This comparative study forms a crucial foundation for future studies of variant genotypes of each of these important pathogenic species.

## MATERIALS AND METHODS

### Bacterial strains and culture conditions.

*M. bovis* PG45 and *M. gallisepticum* Ap3AS were grown aerobically at 37°C in modified Frey’s broth (mycoplasma broth [MB]; 0.75% trypticase peptone, 0.25% phytone peptone, 0.05% proteose peptone, 0.5% yeast extract, 0.5% NaCl, 0.04% KCl, 0.035% MgSO_4_·7H_2_O, 0.005% Na_2_PO_4_, 0.1% glucose, 0.2% DNA, 1% yeast autohydrolysate, 0.0048% phenol red solution, penicillin G at 0.3 mg/ml; pH adjusted to 8.1) containing 10% swine serum (30) for 18 h. For metabolite extraction experiments, both species were passaged twice at 1:10 dilutions, and on the third passage 8 20-ml biological replicates of each species were cultured in 50-ml conical centrifuge tubes (Fisher Scientific) until they reached late logarithmic phase as determined by comparison with previous growth curves. To determine mycoplasma viable numbers, limiting dilution into MB broth in 96-well plates (Thermo Fisher Scientific) was used, with the growth of bacteria within wells determined by observation of changes in the color of the medium, as described previously (14). Briefly, 25 μl of culture was dispensed into each well of the first column of a 96-well plate containing 225 μl of MB medium in each well (Thermo Fisher Scientific). The first column was then 10-fold serially diluted until column 10, with columns 11 and 12 containing sterile broth controls. Test plates were incubated for 3 weeks (*M. gallisepticum*) or 2 weeks (*M. bovis*), before the number of wells in the last 3 columns showing color change were counted, and this number was then converted into the most probable number (MPN) of mycoplasmas present (using an MPN table constructed for eight tubes at three consecutive dilutions) (8). The color-changing units (CCU) per milliter was then calculated by multiplying the MPN by 44.4.

### Metabolic quenching and extraction of polar metabolites.

Late-logarithmic-phase cultures were rapidly quenched to 0°C in an ethanol-dry ice bath to halt metabolic activity. After quenching, mycoplasma cells were harvested by centrifugation (20,000 × *g*, 20 min, 0°C), and the cell pellets were resuspended in ice-cold phosphate-buffered saline (PBS). Cell numbers were adjusted to 1 × 10^10^ CCU (as estimated from previous growth curves), and cell pellets were washed twice with PBS, and centrifuged (17,100 × *g*, 5 min, 0°C) to remove residual culture medium before extraction using chloroform:methanol:water (CHCl_3_:CH_3_OH:H_2_O, 1:3:1 [vol/vol/vol]; 250 µl) containing 1 nmol ^13^C-labeled C-6 sorbitol and 10 nmol ^13^C-6- and ^15^N-labeled valine as internal standards. After vortexing, the samples were incubated at 60°C for 15 min to lyse the cells. Cell debris was removed by centrifugation (17,100 × *g*, 5 min, 0°C), and the supernatant was adjusted to a CHCl_3_:CH_3_OH:H_2_O ratio of 1:3:3 (vol/vol/vol) by addition of distilled H_2_O (dH_2_O) before vortex mixing and centrifugation (17,100 × *g*, 5 min, 0°C) to induce phase separation. The upper aqueous phase, containing polar metabolites, was transferred to a fresh precooled 1.5-ml microcentrifuge tube and stored at −80°C until GC/MS and/or LC/MS analysis.

### Metabolite derivatization and GC/MS analysis.

Aqueous-phase samples were transferred into glass vial inserts and completely dried in a rotational vacuum concentrator (RVC-2-33; John Morris Scientific) at 37°C, with 30 μl of 100% methanol added for the final drying stage. Free aldehyde groups were protected by derivatization in methoxyamine chloride (20 µl, in 30 mg/ml in pyridine; Sigma) with continuous mixing (2 h, 37°C). Metabolites were then derivatized by treatment with 20 μl of *N*,*O*-bis-trimethylsilyltrifluoroacetamide (BSTFA) containing 1% trimethylchlorosilane (TMCS; Thermo Scientific) (1 h, 37°C, with continuous shaking) using a Gerstel MPS2 autosampler robot. For GC/MS analysis, 1 μl of derivatized sample was injected into an Agilent 7890A gas chromatograph (split/splitless inlet, 250°C) containing a VF-5ms column (30 m/250 μm/0.25 μm/10 m Eziguard precolumn) coupled to an Agilent 5975C mass selective detector. Helium was used as the carrier gas at a constant flow rate of 1 ml/min. The GC temperature was ramped from 35°C, at which it was initially held for 2 min, to 325°C, at 25°C/min, and then held for 5 min at 325°C. The 5975C mass selective detector was used in scan mode, and mass spectra data were collected at a rate of 9.19 scans/s over an *m/z* range of 50 to 600 atomic mass units (amu).

10.1128/mSystems.00055-17.9 TABLE S5  Level of metabolite identification for metabolites detected by GC/MS. Download TABLE S5, PDF file, 0.1 MB. Copyright © 2017 Masukagami et al. 2017 Masukagami et al. This content is distributed under the terms of the Creative Commons Attribution 4.0 International license.

### LC/MS analysis of polar metabolites.

Polar metabolites were also analyzed by high-performance liquid chromatography-MS (HPLC/MS). LC analysis was performed on an Agilent Technologies 1200 series HPLC system. Samples were stored in an autosampler at 4°C. Metabolite separation was performed by injecting a 10-µl sample on a SeQuant ZIC-HILIC column (150 mm by 2.1 mm, 5 μm) maintained at 40°C with solvent A [20 mM (NH_4_)_2_CO_3_, pH 9.0; Sigma-Aldrich] and solvent B (100% acetonitrile) at a flow rate of 250 µl/min. The gradients used were time (t) = 0 min, 90% B; *t* = 0.5 min, 90% B; *t* = 12 min, 40% B; *t* = 14 min, 40% B; *t* = 15 min, 5%, *t* = 18 min, 5% B; *t* = 19 min, 90% B.

The mass spectrometry analysis was performed on an Agilent Technologies 6520 series quadrupole time of flight mass spectrometer (QTOF MS). The LC flow was directed to an electrospray ionization source (ESI) where metabolite ionization was performed with a capillary voltage of 3,500 V, a drying gas (N_2_) pressure of 30 lb/in^2^ with a gas flow rate of 7.0 liters/min, a gas temperature in the capillary of 325°C, and fragmentor skimmer cap voltages of 125 V and 65 V, respectively. LC/MS data were collected in centroid mode with a scan range of 50 to 1,700 m/z and an acquisition rate of 1.2 spectra/s in negative MS mode.

10.1128/mSystems.00055-17.10 TABLE S6  Level of metabolite identification for metabolites detected by LC/MS. Download TABLE S6, PDF file, 0.1 MB. Copyright © 2017 Masukagami et al. 2017 Masukagami et al. This content is distributed under the terms of the Creative Commons Attribution 4.0 International license.

Prior to analysis, mass calibration was performed for the negative mode to 0.5-ppm accuracy of the *m/z* value. Internal mass calibration was performed using the Agilent ESI-TOF reference mass solution containing purine (*m*/*z*, 119.036320) and hexakis(1H,1H,3H-tetrafluoropropoxy)phosphazene (*m*/*z* 981.99509), which was continuously infused into the ESI source at a flow rate of 200 µl/min.

### Identification of metabolites.

*M. bovis* and *M. gallisepticum* metabolites were identified from representative chromatograms by using an Agilent MSD Productivity Chemstation for GC and GC/MS. Retention times (RT) and fragmented ion patterns were utilized to identify each metabolite with in-house, Fiehn, Wiley, and NIST libraries, as described previously (31). To generate an untargeted GC/MS data matrix, peak detection and alignment were performed using PyMS (32) (Table S5).

Profiles obtained from LC/MS analyses were filtered to remove the noise peaks above the specific abundance threshold. Metabolites were identified by comparison of retention times and molecular masses with those of the in-house metabolomics Australia library (authentic standards). The remaining metabolites were putatively identified using the MAVEN database, with the molecular mass matching greater than 70% of the mass score match. Peak integration was performed on the spectra from identified metabolites with the in-house library and collated into a targeted data matrix (Table S6).

### Statistical analysis and comparison of metabolite profiles between *M. bovis* and *M. gallisepticum*.

The GC/MS untargeted and LC/MS targeted data matrices were missing value imputed, log transformed, and median normalized across all *M. bovis* and *M. gallisepticum* samples and statistically analyzed using R (MAR, Metabolomics Australia’s in-house R-based statistical analysis package). Missing value imputing refers to the replacement of zeros with either small random numbers between zero and the smallest value of the data matrix or replacement of the zeros with the average value for a metabolite, depending on whether the missing values constitute less than or greater than 50% of the data set for each individual metabolite. This is done to prevent mathematical and statistical errors, such as those that would result from log transformation of zero. To perform median normalization, every metabolites’ peak abundance value was divided by the median value of all the metabolites of an individual sample. This method accounts for any difference in cell number between samples, as it brings all the metabolites and their observed peak values from differing samples to the same base level, in order to perform meaningful statistics. GC/MS data from three of the eight *M. bovis* biological replicates were excluded due to poor chromatography. The metabolite differences between the two species were compared using unpaired Student’s *t* test with the Benjamini-Hochberg adjustment to control for false discoveries, with a *P* level of <0.05 considered significant. Metabolites found to be qualitatively significantly different in GC/MS analyses were identified (as described above).

LC/MS data from one of the eight *M. gallisepticum* biological replicates were excluded from the statistical analysis because of the low density of bacteria in the cultures prior to extraction. The statistical analysis was performed as described above.

### Mapping of metabolites onto metabolic pathways and annotation of novel putative enzymes.

The metabolites detected in each species were mapped onto the predicted metabolic pathways of each species with the KEGG (Kyoto Encyclopedia of Genes and Genomes) pathway mapping tool. The original metabolic maps of the two species were downloaded as KGML files into VANTED (v 2.2.1.) before manual annotation of metabolites onto the metabolic map. We assigned putative functions to enzymes and transporters, using database searches to fill existing gaps between the annotated metabolites on the metabolic map. Predicted protein sequences of *Mycoplasma pneumoniae* strain M129 were predominantly used as the reference for protein BLAST searches against predicted *M. bovis* and *M. gallisepticum* protein sequences. If no gene was annotated in *M. pneumoniae*, then *E. coli*, *Bacillus subtilis*, or other bacterial sequences were used as references (Fig. S3 and S4). Following identification of an orthologous predicted protein in *M. bovis* or *M. gallisepticum*, the sequence was compared with those of other mycoplasmas and other bacteria by using BLASTp (33). The protein structure prediction tools I-TASSER (34) and Phyre2 (35) were also used to assist in predicting protein function.

### ^13^C stable isotope labeling studies.

The [^13^C]glucose labeling studies were conducted with targeted detection of the incorporation of the ^13^C label into polar metabolites. Cultures were passaged as described for untargeted analysis and then labeled for 12 h in MB medium supplemented with either 1 mM U-[^13^C]glycerol or 5 mM U-[^13^C]pyruvate (added to standard MB medium), or in MB medium in which 6.6 mM U-[^13^C]glucose replaced [^12^C]glucose. Cultures were then quenched and polar metabolites were extracted with 1 nmol *scyllo*-inositol as an internal standard and analyzed by GC/MS as described for the untargeted analysis.

Data were analyzed to define the RT and labeled ions of the labeled metabolites by comparison with unlabeled sample data by using NTFD (nontargeted tracer fate detection) software (36, 37). Labeled metabolites were then identified by comparison with in-house standards and libraries on the Agilent Chemstation, as described above. The labeled metabolite spectra were subtracted from those containing the naturally occurring isotope of carbon, and then all metabolites were corrected for naturally occurring background labeling based on the protocol and principles described previously (38). The level of labeling of each metabolite was recorded as a proportion of the total, and these data were used to create a heat map by use of an R script, as described previously (31).

### GC-MS analysis of culture supernatant.

Late-logarithmic-phase bacteria were harvested by centrifugation (18,700 × *g*, 20 min, 0°C), and approximately 1 × 10^10^ CCU was resuspended in 1 ml fresh MB medium in 2-ml round-bottom microcentrifuge tubes. Five 1-ml biological replicates of each species were incubated at 37°C for up to 4 h, and 30-µl samples were taken at 0, 5, 30, 60, and 240 min for quenching and metabolite extraction, as described for untargeted GC-MS analysis of intracellular metabolites. Briefly, cultures were quenched in a dry ice-ethanol bath and centrifuged (17,100 × *g*, 20 min, 0°C), and then 20 μl of supernatant was extracted using chloroform-methanol (CHCl_3_:CH_3_OH, 1:3 [vol/vol], 80 μl) containing 1.2 nmol [^13^C]sorbitol and 12 nmol ^13^C- and ^15^N-labeled valine as internal standards. After vortexing and centrifugation (17,100 × *g*, 5 min, 0°C), the polar metabolites were partitioned by addition of 40 μl dH_2_O. The aqueous phase (containing polar metabolites) was stored at −80°C until GC/MS analysis. Control medium (containing no bacteria) was also incubated and analyzed to assess the stability of compounds over time.

### Data availability.

Metabolomics data have been deposited into the EMBL-EBI MetaboLights database with the identifier MTBLS535.
