# Supplementary material for: Comparative Metabolomics of Mycoplasma bovis and Mycoplasma gallisepticum Reveals Fundamental Differences in Active Metabolic Pathways and Suggests Novel Gene Annotations
Source: mSystems. 2017 Oct 10;2(5):e00055-17. doi: 10.1128/mSystems.00055-17 (PMC5634790; doi:10.1128/mSystems.00055-17)
Supplement: TABLE S1 [file sys005172140st1.pdf]

Table 1. Metabolites identified in *M. gallisepticum* and *M. bovis* and mycoplasma broth (MB) medium analyses.

Compound name	Compound ID	Detection platform		
		M. gallisepticum	*M. bovis*	MB Medium
AMP	C00020	GC/MS*+LC/MS*	GC/MS*+LC/MS*	
ADP	C00008	LC/MS*	LC/MS*	
ATP	C00002	LC/MS*	LC/MS*	
UMP	C00105	GC/MS+LC/MS*	LC/MS*	
UDP	C00015	LC/MS	LC/MS	
UTP	C00075	LC/MS	ND	
Xanthine	C00385	LC/MS*	ND	GC/MS
Xanthosine	C01762	GC/MS+LC/MS	GC/MS	
Thymine	C00178	LC/MS	LC/MS	
dAMP	C00360	LC/MS	LC/MS	
dCMP	C00239	LC/MS*	LC/MS*	
Adenine	C00147	GC/MS*+LC/MS*	GC/MS*+LC/MS*	
Deoxyadenosine	C00559	ND	LC/MS	
Deoxyguanosine	C00330	ND	LC/MS	

Deoxyuridine	C00526	ND	LC/MS	
dTDP	C00363	ND	LC/MS	
dTMP	C00364	ND	LC/MS	
dUMP	C00365	ND	LC/MS*	
GDP	C00035	ND	LC/MS	
Guanine	C00242	ND	LC/MS	
Orotidine-5P	C01103	ND	LC/MS	
Thymidine	C00214	ND	LC/MS	
Uridine	C00299	ND	LC/MS*	
GMP	C00144	LC/MS*	LC/MS*	
CMP	C00055	LC/MS*	GC/MS+LC/MS*	
Guanosine	C00387	GC/MS+LC/MS*	GC/MS+LC/MS*	GC/MS
Uric acid	C00366	GC/MS+LC/MS*	GC/MS	
Adenosine	C00212	GC/MS	GC/MS	
CTP	C00063	LC/MS*	LC/MS*	
Deoxycytidine	C00881	LC/MS*	LC/MS*	
Uracil	C00106	LC/MS*	GC/MS+LC/MS*	

Cytidine	C00475	LC/MS*	LC/MS*	
Pseudouridine	C02067	GC/MS	GC/MS	
Inosine	C00294	ND	ND	GC/MS
IDP	C00104	LC/MS*	LC/MS*	
UDP-D-Galactose	C00052	ND	LC/MS	
UDP-D-Glucose	C00029	ND	LC/MS*	
D-Ribofuranose	C16639 (beta)	ND	GC/MS	
Sucrose	C00089	GC/MS*	GC/MS*	GC/MS*
D-Glucose	C00267/C00221/<br>C00031	GC/MS*	GC/MS*	GC/MS*
Mannitol	C00392	GC/MS+LC/MS*	GC/MS+LC/MS*	GC/MS*
Cellobiose	C00185	LC/MS*	LC/MS*	
D-Erythrose	C01796	LC/MS	LC/MS	
D-Mannose/Talose	C00159/C06467	GC/MS*	GC/MS*	GC/MS*
D-Fructose	C00095	GC/MS*	GC/MS*+LC/MS*	GC/MS*
Arabinofuranose	C06115 (L)	GC/MS	GC/MS	GC/MS
D-Trehalose	C01083	GC/MS*	GC/MS*	GC/MS*

Raffinose	C00492			GC/MS*
Glycerol	C00116	GC/MS	GC/MS	GC/MS*
Myo-inositol	C00137	GC/MS*+LC/MS	GC/MS*+LC/MS	GC/MS*
Allo-inositol		GC/MS	GC/MS	GC/MS
D-Glucose-6P	C00092	GC/MS*+LC/MS*	GC/MS*+LC/MS*	
Sedoheptulose-7P	C05382	GC/MS	GC/MS	
D-Glucopyranose-P		GC/MS	GC/MS	
D-Sorbitol-6P	C01096	GC/MS	GC/MS	
D-Mannopyranose-6P	C00275	GC/MS	GC/MS	
D-Galactonofuranose-6P		GC/MS	GC/MS	
sn-Glycerol-3P	C00093	GC/MS*+LC/MS*	GC/MS*+LC/MS*	GC/MS*
D-Sedoheptulose-1,7P	C00447	LC/MS	LC/MS	
3-Phospho-D-glycerate	C00197	GC/MS*+LC/MS	GC/MS*	
D-Ribose-5P	C00117	LC/MS*	LC/MS*	
Dihydroxyacetonephosphate (DHAP)	C00111	LC/MS	LC/MS	
DL-Glyceraldehyde-3P	C00118/C00661	LC/MS*	LC/MS*	
Octoluse Bisphosphate		LC/MS	LC/MS	

Fructose 1/6-bisphosphate	C00354	LC/MS*	LC/MS*	
Fructose-6P	C00085	GC/MS*	GC/MS*	
2-Deoxy-D-ribose-5P	C00673	LC/MS*	LC/MS*	
Ribulose-5P	C00199/C01101	GC/MS*	GC/MS*	
Glycerate	C00258	GC/MS*+LC/MS	GC/MS*	GC/MS*
Glyoxylate	C00048	LC/MS	ND	
Phosphoenolpyruvate	C00074	LC/MS*	LC/MS*	
(R)-Lactate	C00256	GC/MS*+LC/MS	GC/MS*+LC/MS	GC/MS*
Pyruvate	C00022	LC/MS	ND	
Citraconic acid	C02226	LC/MS	LC/MS	
3-Methylphenylacetic acid		LC/MS	LC/MS	
2-Dehydro-D-gluconate	C06473	LC/MS*	LC/MS*	
Fumarate	C00122	GC/MS*+LC/MS*	GC/MS*+LC/MS*	GC/MS*
Malate	C00711/C00149/	GC/MS+LC/MS*	GC/MS*	
	C00497			
Isocitrate	C00311	GC/MS*+LC/MS*	GC/MS*+LC/MS*	
Citrate	C00158	LC/MS*	LC/MS*	GC/MS*

Succinate	C00042	ND	GC/MS*	
Acetic acid	C00033	GC/MS*	GC/MS*	
D-Galacturonate	C00333	LC/MS*	LC/MS*	
Gluconic acid	C00257	GC/MS		GC/MS
Glucuronic acid	C00191	GC/MS	GC/MS	
D-Galactono-1, 4-lactone	C03383	LC/MS*	LC/MS*	
Urea	C00086	ND	ND	GC/MS*
Ornithine	C00077	GC/MS*+LC/MS	GC/MS*	GC/MS*
Lysine	C00047	GC/MS*+LC/MS*	GC/MS*+LC/MS*	GC/MS*
Arginine	C00062	LC/MS*	LC/MS*	
Leucine	C00123	LC/MS*	LC/MS*	
L-Tryptophan	C00078	GC/MS*+LC/MS*	GC/MS*+LC/MS*	GC/MS*
L-Valine	C00183	GC/MS*+LC/MS*	GC/MS*+LC/MS*	GC/MS*
Aspartic acid	C00049	GC/MS*+LC/MS*	GC/MS*+LC/MS*	GC/MS*
Phenylalanine	C00079	GC/MS*+LC/MS*	GC/MS*+LC/MS*	GC/MS*
Histidine	C00135	GC/MS*+LC/MS*	LC/MS*	GC/MS*
Pyroglutamic acid	C01879/C02237	LC/MS*	GC/MS+LC/MS*	GC/MS

Serine	C00065	GC/MS*+LC/MS	GC/MS*+LC/MS	GC/MS*
L-Proline	C00148	GC/MS*+LC/MS*	GC/MS*+LC/MS*	GC/MS*
Glycine	C00037	GC/MS*+LC/MS*	GC/MS*+LC/MS*	GC/MS*
L-Alanine	C00041	GC/MS*+LC/MS*	GC/MS*+LC/MS*	GC/MS*
Glutathione	C00051	LC/MS*	LC/MS*	
Isoleucine	C00407	GC/MS*+LC/MS*	GC/MS*	GC/MS*
Asparagine	C00152	GC/MS*+LC/MS*	GC/MS*+LC/MS*	GC/MS*
Citrulline	C00327	GC/MS+LC/MS*	GC/MS+LC/MS*	
O-phospho-serine	C01005	GC/MS+LC/MS*	GC/MS+LC/MS*	
L-Threonine	C00188	GC/MS*	GC/MS*	GC/MS*
Homoserine	C00263	GC/MS*	ND	
Methionine	C00073	GC/MS*	GC/MS*	GC/MS*
L-Glutamic acid	C00025	GC/MS+LC/MS*	GC/MS+LC/MS*	GC/MS*
Tyrosine	C00082	GC/MS*+LC/MS*	GC/MS*+LC/MS*	GC/MS*
Taurine	C00245	GC/MS	ND	
D-Glucosamine-1P	C06156	LC/MS*	ND	
2-Keto-isovalerate	C00141	LC/MS	LC/MS	

N-Acetyl-glucosamine-1, 6-P	C04461	LC/MS	LC/MS	
D-Glucosamine	C00329	LC/MS*	LC/MS*	
NADH	C00004	LC/MS*	LC/MS*	
NAD^+	C00003	LC/MS	LC/MS	
FAD	C00016	LC/MS	LC/MS	
$NADP^+$	C00006	LC/MS	ND	
Nicotinamide	C00153	GC/MS+LC/MS	GC/MS	
CoA	C00010	LC/MS	LC/MS	
Lipoate	C00725	LC/MS	ND	
Dephospho-CoA	C00882	ND	LC/MS	
Acetylphosphate	C00227	LC/MS	ND	
Cholesteryl sulfate	C18043	LC/MS*	LC/MS*	
4-Pyridoxic acid	C00847	LC/MS*	LC/MS*	
Ascorbic acid	C00072	ND	ND	GC/MS
Betaine	C00719	LC/MS*	LC/MS*	
Biotin	C00120	LC/MS*	LC/MS*	
Phosphoric acid	C00009	GC/MS	GC/MS	GC/MS

Pyrophosphate	C00013	ND	GC/MS	
Myristic acid	C06424	GC/MS*+LC/MS*	GC/MS*+LC/MS*	
Palmitic acid	C00249	GC/MS*	GC/MS*	GC/MS*
Stearic acid	C01530	GC/MS*	GC/MS*	GC/MS*
Glycerophosphorylglycerol		GC/MS	GC/MS	
2-Oxobutanoate	C00109	LC/MS*	LC/MS*	

Asterisks (*) indicate the metabolites were identified using targeted analysis with authenticated standards on LC/MS and metabolites identified with in house library on GC/MS. The level of identification for each compound, according to the standards proposed by Sumner et al 2007, is listed in Tables S5 and S6. For GC/MS, the fragmented ion and retention time is provided (Table S5), and for LC/MS the retention time, mass accuracy (m/z), obtained mass and ppm errors are provided (Table S6). For metabolites not marked with an asterisk, the level of identification is level 2.
