# Supplementary material for: Comparative Metabolomics of Mycoplasma bovis and Mycoplasma gallisepticum Reveals Fundamental Differences in Active Metabolic Pathways and Suggests Novel Gene Annotations
Source: mSystems. 2017 Oct 10;2(5):e00055-17. doi: 10.1128/mSystems.00055-17 (PMC5634790; doi:10.1128/mSystems.00055-17)
Supplement: FIG S1 [file sys005172140sf7.pdf]

1) Phosphotransferase glucose transporter (PTSG)
Bacillus subtilis used as reference.

```
AIY92692.1|PTSG_BSU13890      MFKALFGVLQKIGRALMLPVAILPAAGILLAIGNAMQNKDMIQVLHFLSNDNVQLVAGVM    60
ADR25276.1|MBOVPG45_0417      ------------------------------------------------------------    0
ACF07467.1|MARTH_orf702       ------------------------------------------------------------    0
AJR12360.1|MDIS_03180         ------------------------------------------------------------    0
AAT27986.1|MMOB5000           ------------------------------------------------------------    0

AIY92692.1|PTSG_BSU13890      ESAGQIVFDNLPLLFAVGVAIGLANGDGVAGIAAIIGYLVMNVSMSAVLLANGTIPSDSV    120
ADR25276.1|MBOVPG45_0417      ------------------------------------------------------------    0
ACF07467.1|MARTH_orf702       ------------------------------------------------------------    0
AJR12360.1|MDIS_03180         ------------------------------------------------------------    0
AAT27986.1|MMOB5000           ------------------------------------------------------------    0

AIY92692.1|PTSG_BSU13890      ERAKFFTENHPAYVNMLGIPTLATGVFGGIIVGVLAALLFNRFYTIELPQYLGFFAGKRF    180
ADR25276.1|MBOVPG45_0417      ------------------------------------------------------------    0
ACF07467.1|MARTH_orf702       ------------------------------------------------------------    0
AJR12360.1|MDIS_03180         ------------------------------------------------------------    0
AAT27986.1|MMOB5000           ------------------------------------------------------------    0

AIY92692.1|PTSG_BSU13890      VPIVTSISALILGLIMLVIWPPIQHGLNAFSTGLVEANPTLAAFIFGVIERSLIPFGLHH    240
ADR25276.1|MBOVPG45_0417      ------------------------------------------------------------    0
ACF07467.1|MARTH_orf702       ------------------------------------------------------------    0
AJR12360.1|MDIS_03180         ------------------------------------------------------------    0
AAT27986.1|MMOB5000           ------------------------------------------------------------    0

AIY92692.1|PTSG_BSU13890      IFYSPFWYEFFSYKSAAGEIIRGDQRIFMAQIKDGVQLTAGTFMTGKYPFMMFGLPAAAL    300
ADR25276.1|MBOVPG45_0417      ------------------------------------------------------------    0
ACF07467.1|MARTH_orf702       ------------------------------------------------------------    0
AJR12360.1|MDIS_03180         ------------------------------------------------------------    0
AAT27986.1|MMOB5000           ------------------------------------------------------------    0

AIY92692.1|PTSG_BSU13890      AIYHEAKPQNKKLVAGIMGSAALTSFLTGITEPLEFSFLFVAPVLFAIHCLFAGLSFMVM    360
ADR25276.1|MBOVPG45_0417      ------------------------------------------------------------    0
ACF07467.1|MARTH_orf702       ------------------------------------------------------------    0
AJR12360.1|MDIS_03180         ------------------------------------------------------------    0
AAT27986.1|MMOB5000           ------------------------------------------------------------    0

AIY92692.1|PTSG_BSU13890      QLLNVKIGMTFSGGLIDYFLFGILPNRTAWWLVIPVGLGLAVIYYFGFRFAIRKFNLKTP    420
ADR25276.1|MBOVPG45_0417      --------MTT-------------GDKWKIATLTIFTLGFCWVYW---SVKNKKVK---K    33
ACF07467.1|MARTH_orf702       --------MSK-------------KNKLKYVLLIIFTLGLICIYW---RFKYRQVE---K    33
AJR12360.1|MDIS_03180         --------MKL-------------ISKIIYFLLQIFSFGFFAKHI---N---KKHN---K    30
AAT27986.1|MMOB5000           --------MSK-------------KDKFLYIFLIIITFGLILLYW---R-KYRQIK---P    32
                                      *.                .:      :   . :*:    :        :: :

AIY92692.1|PTSG_BSU13890      GREDAAEETAAPGKTGEAGDLPYEILQAMGDQENIKHLDACITRLRVTVNDQKKVDKDRL    480
ADR25276.1|MBOVPG45_0417      LKKGEINKLD-------SSMDTLELINLLGGKSNISSVSNTISRIKVIINDKSIVQKDKL    86
ACF07467.1|MARTH_orf702       -KDYLTVEHT-------LAFSFDELMNLIGSKDNIDNVSATQKIIKIYFKERSKVNLTGI    85
AJR12360.1|MDIS_03180         TKSDLTFEKK-------FDFKIEELINLLGGKKNIKSSDFSISRLKIQLKSTENLDFEKI    83
AAT27986.1|MMOB5000           -KKELSISTK-------ISFKLEDLIDSLGSKENIKSIKSSNQIVKIAINDNSLVKIDEV    84
                               :.    .                :::: :* :.**.  .     ::: .:. . :.   :

AIY92692.1|PTSG_BSU13890      KQL-GASGVLEVGNNIQAIFGPRSDGLKTQMQDIIAGRKPRPEPKTSAQEEVGQQVEEVI    539
ADR25276.1|MBOVPG45_0417      EQLKYVSGIMISSNNVSLVVGDYAKKLSEELNNEIAK-----------------------    123
ACF07467.1|MARTH_orf702       QKLPGISGISFQSKAISLVIGNSAKYLEELILKELDFNERS-------------------    126
AJR12360.1|MDIS_03180         KKLKGVSGIVVNSETISLIVGNKSKAISEAINNFE-------------------------    118
AAT27986.1|MMOB5000           KNLKGISGVVNNTTQISLVVGNSAPTIQKELENKLQKS----------------------    122
                              ::*   **:      :. :.*  :  :.  : .

AIY92692.1|PTSG_BSU13890      AEPLQNEIGEEVFVSPITGEIHPITDVPDQVFSGKMMGDGFAILPSEGIVVSPVRGKILN    599
ADR25276.1|MBOVPG45_0417      ------------------------------------------------------------    123
ACF07467.1|MARTH_orf702       ------------------------------------------------------------    126
AJR12360.1|MDIS_03180         ------------------------------------------------------------    118
AAT27986.1|MMOB5000           ------------------------------------------------------------    122

AIY92692.1|PTSG_BSU13890      VFPTKHAIGLQSDGGREILIHFGIDTVSLKGEGFTSFVSEGDRVEPGQKLLEVDLDAVKP    659
ADR25276.1|MBOVPG45_0417      ------------------------------------------------------------    123
ACF07467.1|MARTH_orf702       ------------------------------------------------------------    126
AJR12360.1|MDIS_03180         ------------------------------------------------------------    118
AAT27986.1|MMOB5000           ------------------------------------------------------------    122

AIY92692.1|PTSG_BSU13890      NVPSLMTPIVFTNLAEGETVSIKASGSVNREQEDIVKIEK      699
ADR25276.1|MBOVPG45_0417      ----------------------------------------      123
ACF07467.1|MARTH_orf702       ----------------------------------------      126
AJR12360.1|MDIS_03180         ----------------------------------------      118
AAT27986.1|MMOB5000           ----------------------------------------      122
```

BSU, *Bacillus subtilis*; MBOVPG45, *M. bovis* PG45; MARTH, *M. arthritidis*, MDIS, *M. dispar.*

2) Phosphotransferase glucose transporter (PTSI)
Staphylococcus simulans used as reference.

```
ERS92720.1|PTS_IIBC_SSIM_10465      -------------------------MYNFLQRLGRSLMLPVAVLPAAAIIIGIGNLLKAL
ADR25285.1|MBOVPG45_0573            ------------------------------------------------------------
ADN68838.1|MFE_02260                ------------------------------------------------------------
CAC13190.1|MYPU_0170                MTILKKLNFRKEKSEKSKINNNSGKLKKLLSKISGAFMLPIAIMSIAGIFLGVGAAIATG
AAP56484.2|MGA_0855                 MAS-TTNQVHSQKRSSERWKRFSGHFKEVVGKLSAGIMIPIALLPIAGLLLGIFAAVQNN

ERS92720.1|PTS_IIBC_SSIM_10465      E--V----TPTLAEFIFQSGIAVLEQLGLLFAIGVALGMAKKNDGSVALAAVVGYFVVTS
ADR25285.1|MBOVPG45_0573            ------------------------------------------------------------
ADN68838.1|MFE_02260                ------------------------------------------------------------
CAC13190.1|MYPU_0170                S--GANEGLKLFGLFIQNMGDPIFSSLPLLFAAAFVIAFTEE-AGAAVFAAIIAYLVFSA
AAP56484.2|MGA_0855                 VSQATHPGVFAMAAFFKNGAEAVFGNLPTLFAIAIAVAFTNQ-SGVAALSAFVGWLIFNA

ERS92720.1|PTS_IIBC_SSIM_10465      VLKTE------TLAKILS---------------VKEDSLNIGFTAMNNQNVFIGLIIGLI
ADR25285.1|MBOVPG45_0573            ------------------------------------------------------------
ADN68838.1|MFE_02260                ------------------------------------------------------------
CAC13190.1|MYPU_0170                LQAALIEPLRDSAQKIIGYKVLFESAGRDPESLIRLVGSSVGFTTL-QTSVFGGISVGLI
AAP56484.2|MGA_0855                 TQTALIFPNANSQTFTILYYNSVP---------ATALAMNVGLRSL-STSVFGGVIVGAI

ERS92720.1|PTS_IIBC_SSIM_10465      AAFCYNKFSGTELPTALSFFSGKRLVPIMTAFLSVILTIILLFVWPFVYSGIISFGKWIM
ADR25285.1|MBOVPG45_0573            ------------------------------------------------------------
ADN68838.1|MFE_02260                ------------------------------------------------------------
CAC13190.1|MYPU_0170                TSYLYNKFHKIQFHPAFSFFAGKRFVPLVAILAMVPLSLTFLLFWPWVGKGLSVFGTALG
AAP56484.2|MGA_0855                 TALLFNKFHTIQLPKVVGFFNGNRFVPIITLLTMLPVALVALMIWPAIGIGLNALGQSLG

ERS92720.1|PTS_IIBC_SSIM_10465      DF---GAFGAFLYGFFNRLLIPTGLHHALNSVFWFDLAGINDIAKFQT------------
ADR25285.1|MBOVPG45_0573            ------------------------------------------------------------
ADN68838.1|MFE_02260                ------------------------------------------------------------
CAC13190.1|MYPU_0170                KV--PYGIESFIFGFIERSLIPFGLHHVFYAPLWYSNAGGDAATALSQWQ----------
AAP56484.2|MGA_0855                 VLSRNGNFNSFIFGYVERALVPFGLHHAFYAPLWFTSAGGSIANIIVNQAGSNAIAPLIV

ERS92720.1|PTS_IIBC_SSIM_10465      ---G--------------------------------------------------------
ADR25285.1|MBOVPG45_0573            ------------------------------------------------------------
ADN68838.1|MFE_02260                ------------------------------------------------------------
CAC13190.1|MYPU_0170                --QAGN-------------QFVESAGFTAANIQ-------ELIDSITKNADKWVGDSTGW
AAP56484.2|MGA_0855                 TDENGQRVIRSIVGVAANKDAVKPAGFTAQNWMYWGQVVTALNPNTQINTDSLSGDQRLW

ERS92720.1|PTS_IIBC_SSIM_10465      ------------------------------------------------------------
ADR25285.1|MBOVPG45_0573            ------------------------------------------------------------
ADN68838.1|MFE_02260                ------------------------------------------------------------
CAC13190.1|MYPU_0170                QAVNSLNFNVVSFRKQ--------------------------------------------
AAP56484.2|MGA_0855                 FTINSLFVNKSVYLSGSSDPYTFTFKSFADSTLNHPSLIQSITRNGTVISGAQLLTMIKE

ERS92720.1|PTS_IIBC_SSIM_10465      ----EG-------------AVKGITGRYMAGFFPVMMFGVPAAALAMYQTADSKQKKRVA
ADR25285.1|MBOVPG45_0573            ------------------------------------------------------------
ADN68838.1|MFE_02260                ------------------------------------------------------------
CAC13190.1|MYPU_0170                ---GSSEVQTLRVLDFFAQEFGIKLGRFLQGKYVFMSMGLPAAAFAMVMAAPKENRKVAF
AAP56484.2|MGA_0855                 SSTGSVVLQANDTLNF--AFPGVNPGQYEQGKHAFMIFGLPAAGAAMIMAAPKDQRKYAS

ERS92720.1|PTS_IIBC_SSIM_10465      GLMLAGSISAFFVGVTEPIEFAFMFAAPVLFV-IHALLTGLSLFIAALFHWTAGFSFSAG
ADR25285.1|MBOVPG45_0573            ------------------------------------------------------------
ADN68838.1|MFE_02260                ------------------------------------------------------------
CAC13190.1|MYPU_0170                ASLAPAAFTCFLTGITEPLEFTFLLLSPFLFWGFHAFMCALAFLLMNVLGAHIGMTFSGG
AAP56484.2|MGA_0855                 SIIISAAFTSFLTGITEPIEFTFLFLAPYLFWGFHAVFCAISFWLTSLLGGNIGQTFSGG

ERS92720.1|PTS_IIBC_SSIM_10465      LIDFLLSLINPVS----NHPWMLMVQGVVFFVIYYVVFRVAISVFNLNTPGRGDEKIADP
ADR25285.1|MBOVPG45_0573            ----------------------------------------------MA---------
ADN68838.1|MFE_02260                -----------MG----FVSWI------------------------KRVFGRKDNK----
CAC13190.1|MYPU_0170                FIDYVIYGLIPIQK--GTHAYWVYVVAPFYAPIYFFVFYFWIKFKNLDTPGRGQNIKLFT
AAP56484.2|MGA_0855                 IIDLTIYGFIPDALGAKTNSWLPLVIGLFYIPLYYFTFYFLITKRNIETPGRGAGTRLYT
                                                                                      .

ERS92720.1|PTS_IIBC_SSIM_10465      VDEEGVSSESGKG----------GKYSAMSTQIISGLGGKENITSLTNCATRLRLEVANI
ADR25285.1|MBOVPG45_0573            ----KANKILDTN----------TEEYKQPKQIIQALGGLENISKYRNCATKLRYDIVNK
ADN68838.1|MFE_02260                ----QKIKEVRKL----------VEISQLTRDIVNAMGGIENITGFNNCAVRLRYDVKDT
CAC13190.1|MYPU_0170                KKDF-RNKN-KNK----------NLVDPQVRAVVLGLGGWDNITKYENCASRLRYDVVDF
AAP56484.2|MGA_0855                 KADYQAKVASKNAASTNDGSANFKPIEITSYKLIKAFGGRDNISAVNACATKLRVSVKTK
           .                    :: .:** :**:    ** :** .:

ERS92720.1|PTS_IIBC_SSIM_10465      DQIDKAQIKQSGAAGVTTSGQHNVQVIIGTQVQQVADEVERQL---ESE-----------
ADR25285.1|MBOVPG45_0573            DLVNETELINSGASAVNFVGDNHVQVRFGLKTVKLAKNIREVRDSKSS---KNE------
ADN68838.1|MFE_02260                SLVNEEALKKVGASEVIFIGKRHVQAKFGEISEKLNLDIQEAAPFLEEEALKNKKIEVKS
CAC13190.1|MYPU_0170                SKIDENRIKSAGATAIRRVGSNHIQIIVGSIAEQLNQRVKASIGQSLEK---DSKQSDSN
AAP56484.2|MGA_0855                 EKVNFGEIGALGSLGTYAVSDTLVHAVYGGDADIIKSYMQKMINKDYDSSAIEKVIGDPA
                                    . ::   :   *:      ..  ::   *     :   :.       .

ERS92720.1|PTS_IIBC_SSIM_10465      ------------------------------------------------------------
ADR25285.1|MBOVPG45_0573            ------------------------------------------------------------
ADN68838.1|MFE_02260                DV---NLFEEKPVEVKKT-VDENIVYTPCKGTRKSIEDLSDQTFS--LLGCGYAIEIDKK
CAC13190.1|MYPU_0170                DK---NSTSKANLENPKTQNKNLSVYSVFEGNSHSIVELNDGVFSEKMLGDGVFVEPNEF
AAP56484.2|MGA_0855                 TKEIDPSMPKATPEVDHKLYDVVDVYSPVKGTVTDLADLPDPSFAGKIMGDGVSIEPEDG
```

```
ERS92720.1|PTS_IIBC_SSIM_10465      ------------------------------------------------------------
ADR25285.1|MBOVPG45_0573            ------------------------------------------------------------
ADN68838.1|MFE_02260                LNKLEVYAPINGKLIMSYPTKHAYGIANDNGLEVLVHIGIDTVKLNGLGFETNLKQ----
CAC13190.1|MYPU_0170                KGLQKVYAPVSGKLLTLFETKHAYGITTNEGIEILVHIGVDTVNLKGQGFKTNLVQ----
AAP56484.2|MGA_0855                 ----MFYSPVDGKLELAYNTGHAYFFD-VNGAKILIHIGIDTVSLNSNNSDSNNLVGFKM

ERS92720.1|PTS_IIBC_SSIM_10465      ------------------------------------------------------------
ADR25285.1|MBOVPG45_0573            ------------------------------------------------------------
ADN68838.1|MFE_02260                ----NDKVERGQ-LLATVDLKKLRNSKICSDVIVLITDNVNKNKQTKLL-CPLEIKEVNV
CAC13190.1|MYPU_0170                ----GQDVKIGD-VLAEVDYDFLSKQNLPSMSTIVVITNMEQSKITNLKQGPVAL---NQ
AAP56484.2|MGA_0855                 FAKSGDNVSFKKSPVVQANLEMIKKKELSTNSPILALKETLANYDLKVVVKPGDKVNVGD

ERS92720.1|PTS_IIBC_SSIM_10465      ----------
ADR25285.1|MBOVPG45_0573            ----------
ADN68838.1|MFE_02260                PWFKMVD---
CAC13190.1|MYPU_0170                KLFDLEQN--
AAP56484.2|MGA_0855                 LLFKLTKKNK
```

SSIM, *Staphylococcus simulans*; MFE, *M. fermentans*; MPYU, *M. pulmonis*; MGA, *M. gallisepticum*

3) Pfk family carbohydrate kinase (PFK)

Clostridium sordellii used as reference. Active site is not known but substrate binding sites are shown in red.

```
CEQ19350.1|PFKB_W2948_29021_CLOSR      MTDREKEILNILIEDPMVSQQELADKLNITRSSVAVHITNLMKKGHIKGKGYIINKGNFV    60
ADR25403.1|MBOVPG45_0026               ---------------------------------------------------------MSV    3
CCO56448.1|VIBNI_A0244                 MTEREQQIFQLIKQDPMIPQQDIADKLGISRSAVAGHIMNMTNKGIILGKGYILSESQYA    60
WP_014584243.1|HF1_14770               -----------------------------------------MK--------------KSI    5

CEQ19350.1|PFKB_W2948_29021_CLOSR      TVIGGSNIDIQGSPNNTL-------------------TMFDSNPGKVDISLGGVGRNISE    101
ADR25403.1|MBOVPG45_0026               VVVGSINYDLYAKVNNKI-------------------SLRDSNPANIYSSLGGVAYNIAK    44
CCO56448.1|VIBNI_A0244                 VVIGGANMDILGQPQQAM-------------------HQGDSNPGVVSCSPGGVGRNIAE    101
WP_014584243.1|HF1_14770               LCLGNPVFDTFASVSDDILVSNNIEKGSAPILDPKKVIEIISKVNFISANCGGSASNIAK    65
                                         :*    *  .. .: :                       *:   :  . ** . **::

CEQ19350.1|PFKB_W2948_29021_CLOSR      NIAKLGLNTKLLSAVGNDIYGNKILSECKQLNIDVSDCYVSDAFPTSMY-----------    150
ADR25403.1|MBOVPG45_0026               NLALSGIDTTLISAVGNDLEANWIKKQLENDSLN-SILIAKNDYKTAKY-----------    92
CCO56448.1|VIBNI_A0244                 NIARLGTEVRLISVVGNDTYGQLIIDKTRQAGVDVSPVMKLSDGTTSTY-----------    150
WP_014584243.1|HF1_14770               GIALVGGKAGLFGQYADDKEGDIIKDSLKEHGVI-DHCSVEKGGITTQINCLVTPDAQRT    124
                                        :*  * .. *:.  .:*  .: * .. .:  :  .     .   *:

CEQ19350.1|PFKB_W2948_29021_CLOSR      -LSILNSDYDMQLAISHMDIIEKIDIDYIVSKHNSIKDSLAIVLDTNLSYEVIDYISRTY    209
ADR25403.1|MBOVPG45_0026               -VALIDNNSDMNVAASDTKITESIDYNDLFAYKKLLDKSTYLCLDANLSSYLIVKLCKNF    151
CCO56448.1|VIBNI_A0244                 -LSLLNEDGDMQVAVSDMSILDKLTIESLEPHIEVLKRAEVIVVDTNLSESVLEYIFSQF    209
WP_014584243.1|HF1_14770               MIPLFGASHFMNPEAVDYSVVDNYDYFLLEGYQFCNQ-----CL-VDISYAFLDRVKEKN    178
                                        : ::  .  *:    . .: :.     :       .      : .::*  .:  :

CEQ19350.1|PFKB_W2948_29021_CLOSR      TNIPIFIDTVSTQKSLKI-------KDIIGRFNTIKPNKLEAEILSDIKINTQDDLKQCA    262
ADR25403.1|MBOVPG45_0026               SDKFIVAEAVSSQKILKF-------KPVLKHISLLKGNKLEFQALLNT---SETDLFILA    201
CCO56448.1|VIBNI_A0244                 ADKPIFVDTVSSTKAVKI-------KPYLSHIHTLKPNIKEAQSLSDLPHHDADDLETIA    262
WP_014584243.1|HF1_14770               IS-----LILSISNIFCVESYYQHMKHFAEAARMIVGNEEEFLKLFNF-----DDVNKLL    228
                                        .       :*  : . .       *        :  *  *   * :       *:

CEQ19350.1|PFKB_W2948_29021_CLOSR      QYF----LEK-GVKNVFITLGQDGVFCANENESLLL-RGVKVNAVNATGAGDAFISALVY    316
ADR25403.1|MBOVPG45_0026               SKV----LEL-GIKSVVITDGENGCIYANNEGVVEFYRPTIVKALSTSGAGDAFCAGLIY    256
CCO56448.1|VIBNI_A0244                 DWF----LNQ-GTQRIFLSLGEEGVFYKDAQGSQHF-PTVSTDIKNANGAGDAFLAGLVH    316
WP_014584243.1|HF1_14770               DHLQSQCVKGGKYEMIMVTAGPKGANILWEGKRFFVEAPDVKTPVDTTGAGDYFVAGLLY    288
                                       . .    ::    : :.:: * .*           .         .:.**** * :.*::

CEQ19350.1|PFKB_W2948_29021_CLOSR      SYLNHFNLKDTAEFSTAASILTLLHKD-TINPSMSVDSVEKILKEMI-       362
ADR25403.1|MBOVPG45_0026               GLINKMQILKVGASF---AKFSLLSLM-TINDKLNKSSLSKELMEVN-       299
CCO56448.1|VIBNI_A0244                 SYINKWSVERSSEFAMAAASVALSDVA-TINPNFSENAVKRVLNEAEC       363
WP_014584243.1|HF1_14770               GYFQGYDMSISNKIAQIMAKDIISKFGSTLSPS-VVDEVKKVLP----       331
                                       . ::  .:          :   :     *:. .   . :.: *
```

W2948_29021, *Clostridium sordellii*; VIBNI, *Vibrio nigripulchritudo;* HF1_14770, *M. haemofelis*

4) Acetaldehyde dehydrogenase (ADH)

M. pneumoniae used as reference. No known active site.

```
NP_110253.1|ADH_MPN564        MKAYAMLKIGAT----GWIEKPRPVCGPNDAIIRPLAVAPCTSDVHTVWEGGIGERHNMV        56
ADR25320.1|MBOVPG45_0543      MKMKALVYHGDHKIALETVDK-PVILKPTDAIVKVTKTTICGTDLGIYKGKNPEVADGRI        59
                              **  *::  *        ::*   :  *.***::   .: * :*:           .  :

NP_110253.1|ADH_MPN564        LGHEGCGVVDEVGSEVKSFKVGDRVLVAAITPEWNSVNAQAGYPMHSGGMLGGWKFSNVK        116
ADR25320.1|MBOVPG45_0543      LGHEGIGIVEEVGSSVSNVKVGDKVLIGCVTPCGKCDNCRQQLYSHCREAEGGWKFGYMI        119
                              ***** *:*:****.*...****:**:..:**  :. *.:     *.    *****. :

NP_110253.1|ADH_MPN564        DGMFAEYFHVNDAEGNLALMPEGMDLADACMLSDMIPTGFHAN-ELADIQYGVALSFFCA        175
ADR25320.1|MBOVPG45_0543      NGTQAEYVRVPFADNSLYKYPESISDEVAVMLSDALPTGHEIGVQYGNVAPGKSVAIVGA        179
                              :*  ***.:*  *: .*   **.:.   * **** :***..   : .::  * ::::. *
```

```
NP_110253.1|ADH_MPN564        GPVGLMAIAGAALKGAGRIIVVDSRPDIVEIAKAYGATDYIDFKKVSVVDEILKWTNNEG      235
ADR25320.1|MBOVPG45_0543      GPVGMGALLTAQLYSPAHLIVIDFDKNRLEMAKKLGATHTLVPDET-LLTKLQEIVGTDG      238
                              ****: *:  * * . .::**:*   : :*:**  ***. :  .:. :: :: : . .:*

NP_110253.1|ADH_MPN564        VEKVLISGGGSTILETAIKVLRPGGKIGNVNYFGAGEFLTIPRVEWGVGMAHKAIHGGLM      295
ADR25320.1|MBOVPG45_0543      VDVAIEAVGVPQSWDTCQKIVKAGGNISVVGVHGKKVDFNVQE-LWIKNL---TVTTGLV      294
                              *: .: : *     :*. *::: **:*. *  .*    :.: .  *   :   ::  **:

NP_110253.1|ADH_MPN564        LGGRLRLEKLARLIMTKKLDPSKMITHRFKGFEHIEEALFLMKDKPKDLIKSVVIF----      351
ADR25320.1|MBOVPG45_0543      NTN--TLPMLINAVSTGKLPVQGLITHRFNLSDMMKAYDTFLNASDN---KAMKIFIDAT      349
                                    *  * . : * **  . :*****:  : ::    ::: . :   *:: **

NP_110253.1|ADH_MPN564        -       351
ADR25320.1|MBOVPG45_0543      K       350
```

MPN, *M. pneumoniae.*

6) 5'-nucleotidase (NT5; USHA)

E. coli used as reference.

```
NP_415013.1|USHA_b0480_ECOLI      ------------------------------------------------------------      0
ADR25153.1|MBOVPG45_0690          MKRKFILGLGA-ISAFAAIPMVAATCGVSSPK-----------------QVAKARAAYDE      42
ENY68833.1|MAU_3200               MKKKMLLSLGGILTAFSALPMIAISCGQPNEE-----------------KVAKAKAAYEN      43
ENY69482.1|MBVG_3940              MKKRLFLNLMP-VAALSMLPTIAISCINTKQKDKD---------TQSNTQFEVQLAEYEK      50
ADE19785.1|MCRO_0158              MKIKKLMLVGAIAPLVILPATISAACDDKNKPVKPVTPSTNEMTAEEKKALDAARVEFKD      60

NP_415013.1|USHA_b0480_ECOLI      --------------------------------------------------MKLLQRGVAL      10
ADR25153.1|MBOVPG45_0690          AFKKFNDAVESDIKELVQKKEAINAKDIDKEKKKKLTEEKDAWVKTRTPILQGLRKKAND      102
ENY68833.1|MAU_3200               AFKNFDEAINADMQSLKDKKAEIE-KEKDETKKKELIAKKEEFVKQRSPILLALRKKVND      102
ENY69482.1|MBVG_3940              HYKLFNDTISKYKKELEEKYKNIQKRGNTKEQTKQLTAKKDAWVKEKTIELQALRKNVDD      110
ADE19785.1|MCRO_0158              AMGNYNAQLLTFSKELAAKKKAID-KEKDKTKKEALQKERKQFVAEKSKIVAPLREEINK      119
                                                                                    :  *:.

NP_415013.1|USHA_b0480_ECOLI      ALLTTFTLASETALAYEQDKTYKITVLHTNDHHGHFWRN--EYGEYGLAAQKTLVDGIRK      68
ADR25153.1|MBOVPG45_0690          AFINL-------RKVQKNFKTQVVKVVHTNDEHGRLVFDDGKYNNYS------GMQGLAE      149
ENY68833.1|MAU_3200               AYKNL-------RVAQKGAGTQLIKIVHTNDEHGRLVFDDGKYNNYS------GMQGLAE      149
ENY69482.1|MBVG_3940              NYLKL-------RNLK-DHKTRLVKLIHTNDEHGRLVFDNGKYNKYS------GMQGHAE      156
ADE19785.1|MCRO_0158              ALVKV-------NKLEAKSDTQTIKIFNTNDEHGRLVFDDGKYNNFS------GMDTLAK      166
                                     .                *  :.:..***.**::  :  :* ::.       ::   :

NP_415013.1|USHA_b0480_ECOLI      EVAAEGGSVLLLSGGDINTGVPESDLQDAEPDFRGMNLVGYDAMAIGNHEFDNPLTVLRQ      128
ADR25153.1|MBOVPG45_0690          VLNKDFDRDLLLSAGDLIQGLPLSDSDKGLTISKVAKEMKYQAVAIGNHEFDYGLEHMFN      209
ENY68833.1|MAU_3200               ILSRDFDRDLLLSAGDLIQGLPLSDSDKGLTISKIAKEMKYNAVAIGNHEFDYGLDHMFK      209
ENY69482.1|MBVG_3940              ILGLNFTRDLLLSAGDLIQGQPLSDSDKGVTISEIAALMNYNAVAVGNHEFDYGLMHLFE      216
ADE19785.1|MCRO_0158              FM-QNVEHSLLLSAGDLIQGLPMNDSDKGVTISLVAKEMNYDAIAIGNHEFDFGLEHMIN      225
                                   :  :    ****.**:  * * .* :..         : *:*:*:******  *  : :

NP_415013.1|USHA_b0480_ECOLI      QEK--WAKFPLLSANIYQK----------------STGERLFKPWALFK-RQDLKIAVIG      169
ADR25153.1|MBOVPG45_0690          IEKQT-AGMPFLSANIVWNDKAVAEKVKTKDGKLAVKNEQVFTPYIIKTLESGIKVGIMG      268
ENY68833.1|MAU_3200               IEKET-EGMPFLSANIVWNKKAVTDQVKDKNGKLAIEGQKVFSPYIIKELPSGLKVAIMG      268
ENY69482.1|MBVG_3940              IQKKT-PNMPFLSANIVWNQKAVTDKVKDIDGKDAVADELVFKPYIIKELASGIKVGIMG      275
ADE19785.1|MCRO_0158              IDKQTKDKMPFLSANVVYKDTMP----------EGVKGTRPFTPYKVVTLANGFKVALIG      275
                                   :*     :*:****:  :                      *.*: :    . :*:.::*

NP_415013.1|USHA_b0480_ECOLI      LTTDDTAKIGNPEYFTDIEFRKPADEAKLVIQELQQTEKPDIIIAATHMGHYDNGEHGSN      229
ADR25153.1|MBOVPG45_0690          ITTPDTQWTSNPKNSVHVTFLDPIESGKKTVEELKSK-GVNFIIALTHLGVNRPDTRWDS      327
ENY68833.1|MAU_3200               ITTPDTQWTSAPRNSVHVTFLDPIDTAKEVVKELKAK-GINFIVALTHLGVNRPDTRWDS      327
ENY69482.1|MBVG_3940              ITTPDTAWTSSPKNSVHVTFKDPVQEGTKYAKELRDK-GVNIVIALTHLGVDRQNKEWDS      334
ADE19785.1|MCRO_0158              ITTPDTTITSHPRNSKDVIFKDPVTAAKEIVTEIENKEKINFIIALTHLGVGRNQVEWDS      335
                                  :** **   . *.   .: * .*   ..    *:. .   ::::* **:*      . ..

NP_415013.1|USHA_b0480_ECOLI      APGDVEMARALPAGSLAMIVGGHSQDPVCMAAENKKQVDYVPGTPCKPDQQNGIWIVQAH      289
ADR25153.1|MBOVPG45_0690          R----EF--TKNVAGVDLVLDGHSHTRVDIE-----------------RKDEEKGFLTQAE      365
ENY68833.1|MAU_3200               R----EF--TKNVEGIDLVLDGHSHTLVPIE-----------------EGKDENGFLTQAE      365
ENY69482.1|MBVG_3940              R----TF--ANNVEGVDLVLDGHSHTLVKAE-----------------K--INNTILTQAD      370
ADE19785.1|MCRO_0158              R----YF--ADNATGVDLTIDGHSHTKVDLE-----------------K--NKESWLTQTE      371
                                        :  :  . .: : : ***:  *                       :   :.*:.

NP_415013.1|USHA_b0480_ECOLI      EWGKYVGRADFEFRNGEMKMVNYQLIPVNLKKKVTWEDGKSERVL--------YTPEIAE      341
ADR25153.1|MBOVPG45_0690          CYTKYLSELDLVVDKETGKVTEVKQY-LRTIEYTELLGGKENSIASIKDMIGKLKEKFDA      424
ENY68833.1|MAU_3200               CYTKYLSELDLIVNTKTGKISSVKQR-LWTIEYTELMDGKKNTITPIKNMIDALKTKFDA      424
ENY69482.1|MBVG_3940              SYTKYLSELDIYIDTETNKITKVNQQ-LRTMEETELLGGAVNNNKIIDEKITKLKAEFEK      429
ADE19785.1|MCRO_0158              CYTKYLSEIDLLVDKKTGKIVENLGQVQRDINEVEIA-SHGKENKKVDELIAALTKKFGE      430
                                   : **:.. *: . .   *: .         : .    .  :            . ::

NP_415013.1|USHA_b0480_ECOLI      NQQMISLLSPFQNKGKAQLEVKIGETNGRLEGDRDKVRFVQTNMGRLILAAQMD------      395
```

```
ADR25153.1|MBOVPG45_0690          VNDKVVFNLPIEFKH--TEEVKIKPGNGEKEVSIWRGRAQQTNLGTFAADAAAYDFIENN    482
ENY68833.1|MAU_3200               VNNEVVFSLPFDFKH--TEEVKIKPGNGSKEVPIWKGRSQQTNLGSFTTDATAWDFIENS    482
ENY69482.1|MBVG_3940              INGVKVFDNPIDFIH--TTSHKVEEANKS----SALGRQKQTNLGMFVADALGWSFISDK    483
ADE19785.1|MCRO_0158              VNNVLAFKNTVHFKH--IENIKVD------GIEFWRGRVEQTNLGVLAANAIADELAKAK    482
                                   :    :   ..        . *:               *  ***:* :   *

NP_415013.1|USHA_b0480_ECOLI      ----------RTGADFAVMSGGGIRDSIEAGDISYKNVLKVQPFGNVVVYADMTGKEVID    445
ADR25153.1|MBOVPG45_0690          PKVNGQVVQFNADNVIGLVNGGGLRQDTQSGDIKRADLLGISPFGNRIAAVQVKGSVLLE    542
ENY68833.1|MAU_3200               PKVNNEAVVFNADNVIGLEVGGALRQDTQKGAIKRADLLGIAPFGNRIAAVQVKGEVLLE    542
ENY69482.1|MBVG_3940              STADGQ--KYNLDNTIAFVGGGSIRENLVKGPITRGNMFAVSPYGNRIAAVELKGDKLIE    541
ADE19785.1|MCRO_0158              KDTKDFDKYYSEDKMIGLMNGGGLRADLPVGDVKRGDVLGVLPFGNRIAAVRVDGQTLID    542
                                              :..  **.:* .    * :.  ::: : *:** :. . : *. :::

NP_415013.1|USHA_b0480_ECOLI      YLTAVAQMKPDSGAYPQFANV-SFVA------KDG--------KLNDLKIKGEPVDPAKT    490
ADR25153.1|MBOVPG45_0690          AMKHG-ASKVFSGAYAQYSHNVSAKISYKET-KDG--KFIYELDEDSVMINNKKVEKDKD    598
ENY68833.1|MAU_3200               AMKHG-AKKIFSGGYAQYSHNVSAKINFKEE-KDN--KFVYELDESSVKINGKSVNKDQN    598
ENY69482.1|MBVG_3940              VVAHG-AKKIFSGGFAQWSKNVKLDINFDPT-D-----KIYKPVEESIKINDKAIDRNAT    594
ADE19785.1|MCRO_0158              AIKHG-ASKVKSGAYPQFSQNVSFTIKKTETIKDGKKVYTYEADESTIKIHDKEINKTET    601
                                   :       *  **.: *::.  .           .             . : *. : ::

NP_415013.1|USHA_b0480_ECOLI      YRMATLNFNATGGDGYPRLDN---KPGYVNTGFIDAEVLKAYIQKSSPLDVSVYEPKGEV    547
ADR25153.1|MBOVPG45_0690          YYIVTNDFILIGGDGYEMLDYVKHPE-KAKSVFEGGDILESYIKFGQLITNKEQ-K--ES    654
ENY68833.1|MAU_3200               YYIVTNGFVLEGGDGYDMLNYTKNPT-KAKSVFEGGDILETYIKYGKLITSSNN-T--NS    654
ENY69482.1|MBVG_3940              YYLVTDDYILVGGDEYNMLNYAKITDGSVKLIFEGSDMLQELIKYGQHITKTDF-KA-TE    652
ADE19785.1|MCRO_0158              YTIVTNDFILAGGDGYKMLNFMENK--KATTDYEGGDLLEVMINHFKYSTDSTNFAEGKT    659
                                  * :.*  :   *** *  *:          ..  :  .::*:  *:  .     .

NP_415013.1|USHA_b0480_ECOLI      SWQ---------------------------    550
ADR25153.1|MBOVPG45_0690          TNAFGKRKVDDYKNI-SPNNIEVKHSK---    680
ENY68833.1|MAU_3200               SNPFAKYKTDDYKDP-NPTYIQVNHNKK--    681
ENY69482.1|MBVG_3940              NNPFGDAQIDFYNQEKLPKNVVVNHKAPAL    682
ADE19785.1|MCRO_0158              NTPFA-HELSYYNKPEILTKIKIEG-----    683
                                  .
```

ECOLI, *E. coli* ; MAU, *M. auris,* MBVG, *M. bovigenitalium;* MCRO, *M. crocodyli*

7) Amino acid permease

Lactobacillus crispatus used as reference. No known active site.

```
EST03631.1|Lc367_0497_LACR      -------------MDNKLKRSIGFGSAFSIVIGTIIGSGIFFKQASVLDS-AGSTTMALL     46
ADR24969.1|MBOVPG45_0533        ------MSKKSDAIADNNSKKISFISAMLITVGSSFGAGIFFKSKSVLDSSHGSLILAIF     54
NP_109996.1|MPN308              --------------MKQQKPKISFIAAMLIVIGSSIGAGIFFKSSTVLENSQASLVLAIF     46
ENY68956.1|MBVG_6250            ---MTEQNSQSNFKNAPKHKKMGFFSAMLIVMGSSIGAGIFFKSEEVLNNSRGNLVLAII     57
AAP56893.2|MGA_0287             MPIIRMNKKNRSLKDAPKSKKIGFFSGITIAIGSSIGAGIFFKAQAVLQNSHYSLAFAIF     60
                                                     .:.* :.: *.:*: :*:*****   **:.   .  :*::

EST03631.1|Lc367_0497_LACR      AWIFGGLITLAAGLTIAEIGAQMPYTGGLYVYIENLYGR-----ILGFMAGW------MQ     95
ADR24969.1|MBOVPG45_0533        AWIIASVAVICIALALVEISSTHSDNLSLTSWNKVFNNRFVYNVSKDFNTYVCLPLVFFF    114
NP_109996.1|MPN308              NWLVASVAVIAMALALIEIASVRNDNLSIISWVKVFNRRWLYHGCKNFMTYLYLPLTFFF    106
ENY68956.1|MBVG_6250            CWIIASFSVISMAMAIIEVAGVKNDNLSLIGWNRIFNSRWVHQASKNFMVYLTLPLTFFY    117
AAP56893.2|MGA_0287             SWLFAAFSVTSMALALVEISSGRNDNLSIIGWCQTFNNHYVYKTCKNFMVYIYVPLTYFF    120
                                 *:.... . . .::: *:..     . .:  : . :  :         * .        :

EST03631.1|Lc367_0497_LACR      IIVYG-PAIIAAVAGFMSIMMTNFFGLNHSWRIPLALITVLAIG--IMNFFENKVGAIFS    152
ADR24969.1|MBOVPG45_0533        IPLYSFISLQDGLKFLT-NKT--GFGTGHDWVIWMFISLALTLYFLTAPALYSKVGDIQN    171
NP_109996.1|MPN308              MPLYFICSIQDGFRGLLGLETGAHFNTSVDWLIWLALALIITTYFLTIPPLYAKVGNIQN    166
ENY68956.1|MBVG_6250            MPVYTIMVLQDGLGALA-NKEALTFGTNNDWLIWLAITTFIGLYFLTIPTLISKIGNIHN    176
AAP56893.2|MGA_0287             MPFFFLLSIQDGIRGFNENYH--GLNTKADWAILMLVSVGISAYFIVVSGISSSAANFQN    178
                                : .:    :  ..  :          :    .* * : :   :          :  . . : .

EST03631.1|Lc367_0497_LACR      VITTIAKMIPIAVITICGICWGGQDAINQTVAK----------------------VSQH    189
ADR24969.1|MBOVPG45_0533        IISLAIKLVPLIFITFLGFILVATGKGGASEVSLLVTK------NHSYIEFIKQGKGIEH    225
NP_109996.1|MPN308              MVVSAVKFIPLVFVPIIGFIVAGTGNGELKNVKALVQPPQINGATASFTQLVQAGYGITR    226
ENY68956.1|MBVG_6250            IIVLCIKFLPLVFIIIIGIVLAVTGKGGTDSVKILEFE------KN---YNIKTGSSIIH    227
AAP56893.2|MGA_0287             LIISVVKFLPLLLAIILGFVIFNHNGSKPVNPKIGL------GFQNSATTDLA---TKYS    229
                                ::     *::*: .  : *:               .

EST03631.1|Lc367_0497_LACR      TSNFG------VAVLATLFGYDGWILIANLGGEMKNPQKLLPRAIIAGITAVLAIYTLIT    243
ADR24969.1|MBOVPG45_0533        FNHIGAGLGMFLAIASIFYAYDGFYVSAGIQSEMKEPKKT-PMALFLGLSLTTLIYLIIA    284
NP_109996.1|MPN308              FTGIGAGMGSFISIAAIFFAYDGFYVTAGLQSEMREPKKT-PWALFLGLLITTLFYLILA    285
ENY68956.1|MBVG_6250            YGGFGGFIGIFLSIGAIFFAYDGFYIAAGMQSEMKNPKKT-SLALSLGLIIITIIYLLIA    286
AAP56893.2|MGA_0287             FSNLSPGFGLFISVGGIYFAYDGFYYAAGIQTEMKKPQKT-PLAILIGLLFVTSVYLIMA    288
                                   :.       ::: .  :.***:    * :  **::*:*     *:  *:      .* :::

EST03631.1|Lc367_0497_LACR      IGIFRFIPANQIHQLGENTTTYLANAVFGAIGGKILSIGIIISMMGTLNGKMLTFPRIVY    303
ADR24969.1|MBOVPG45_0533        ISMSINGGSFS--KMHAYSINLMGL-KATNIIFGIMNIFIAIGVLGIANGFAMWMPRYIE    341
NP_109996.1|MPN308              VALSINGGLFS--GMEESMGKLFNNKRAGQIVFGVVNLMIGIGVLGIINGFALWAPRFVE    343
ENY68956.1|MBVG_6250            ISMSINGGSFS--QMRQFIENIMGN-KTGRIIFGLINIAIAIGVIGVINGFSMWMPRYIE    343
AAP56893.2|MGA_0287             IAMSLGSTDGSPFGFKAYFA-----RNDLLPLYAVFQITIGVGILGIINGLSLWANRFME    343
                                :.:         .    :                    :..: * :.::*   **   :    * :
```

```
EST03631.1|Lc367_0497_LACR      AMAKRGDLPFSKYLAYVTPKGKSPVVATLFIVLLALLMMIFFDP----------------      347
ADR24969.1|MBOVPG45_0533        DLLIKGDLPFWEKLAPKVNP-RKPVVGIIYSSIISVPLIIIFTLIGALGYIDTSSYGTVY      400
NP_109996.1|MPN308              DLLAQGDLPFWKQVQGRLNP-NKPVVGVIYCLVLSLTVQVLFTVIGALAYLPTVADYKNY      402
ENY68956.1|MBVG_6250            SLLAEGELPFWRLTIKKLNP-NRPVIGVIYSLIIGLSANIIFTIIGAKLYLPTNDIYLKY      402
AAP56893.2|MGA_0287             DLIKKFELPFSMKLINKISP-NKVVVGMQYNLILAVPVVIIASLIGGLGYIDNSYSDADY      402
                                 :  . :***           .  *:.  :  ::.:   ::

EST03631.1|Lc367_0497_LACR      ------------------DHLSDLCVFTVYCFYLLAFFGIFILRKKNKAERPFSTPLYPF      389
ADR24969.1|MBOVPG45_0533        -----DSTMSMAKLYSFADLMANWNALGCFLLVALAIYGGIRNRKTNKVEIPSKKKYFLP      455
NP_109996.1|MPN308              VNTEIDKLNSMQWLYSFSDLMATWTSLFTFAFIACAIFGAIVNRKTKKITIANPKRYFLP      462
ENY68956.1|MBVG_6250            -----G--TDMASIYSFTDLIANWITVFTFVFIALAIIGALRNRKTKKVPISNPKKSFKF      455
AAP56893.2|MGA_0287             -------GTGVGRIYSFSDLMANWTSVIAFVYIVIAIMGGLANRKTNKIKVN-KSKIFVP      454
                                                  * ::    .  :     *: * :  **.:*      .  :

EST03631.1|Lc367_0497_LACR      VPIIAILGGLFVLISELINDPAGVLL----------------------------------      415
ADR24969.1|MBOVPG45_0533        TAWISILFVASAIFISILVPIINLFLLIGIDRSL---ISN-------VEFTQLLVGRLML      505
NP_109996.1|MPN308              AAWIAVVVNCISVFVTIIEPFINLFLLFGYDETVAHTVL----GNDFIELNELVIGRVML      518
ENY68956.1|MBVG_6250            FAYCTIIFVSLAMLAQILVPILDFALIFVFDIKQYAIEKNVSYEQAQNFFISLAISRSML      515
AAP56893.2|MGA_0287             SAVCAIFTMVVSVSLTFFQPMADALLLYNIPF-------N-------DQYKNVYISRILL      500
                                    ::.     :   ::       *

EST03631.1|Lc367_0497_LACR      ------FAGIVIIGLPIFYAVKRMNKHRLH------------------------------      439
ADR24969.1|MBOVPG45_0533        IVVLILFVSLSILPTIIL---NKIRTKKFGSIDKYYEYTEEKLKSLK---TRLSTIN---      556
NP_109996.1|MPN308              IVVLVFFAIISFLPVYVE---DQYHKRKFGSLANYQQYVQQHLAHST---ING-------      565
ENY68956.1|MBVG_6250            VAVLIIFHFLVFLPIPIF---DKYNKRKYGSLEAFEQYKANFIKTNL---RKNDA-----      564
AAP56893.2|MGA_0287             VLVLFVFLFIMFIPILIE---DQLMIKRYGSVSKGELAKLRIKAEVKKTSFKQELLDYIE      557
                                      *  : ::   :    .:   ::

EST03631.1|Lc367_0497_LACR      --------------------------- 439
ADR24969.1|MBOVPG45_0533        --------------------------- 556
NP_109996.1|MPN308              --------------------------- 565
ENY68956.1|MBVG_6250            --------------------------- 564
AAP56893.2|MGA_0287             TLKTKDLNDDLKAALKEVNVEVDELII 584
```

LACR, *Lactobacillus crispatus*.

8) Glycine cleavage H protein (GCSH)

Aerococcus urinae used as reference. No known active site.

```
AEA00776.1|HMPREF9243_0576_AEUR      MTKRGNYLFVEKNG--DLYTVSMTPELQDDIGTVGYAEFAQED--QVEKDDALLNIEASK 56
ADR24909.1|MBOVPG45_0459             MKKLIGKFTLEYLDGKNEYVLRLTPDAQDELISIKELKILVKENSAYSTDELFMIIHTAK 60
WP_004420912.1|MBVG_4610             MKKIVKYLIVEKLETKDQYYLRMTPEMQDDIGTVGHIQFRNTDKSMLKENDEFMAFEASK 60
ACU79130.1|MMCAP1_0466               MKKVIKFLVIEKIDDKNEYYLRMTSEMQDDIGTVSYLQFKNTDKKHLKEDDIFLALEASK 60
AGJ91024.1|MPUT9231_6310             MKKVINYLVLEKQENSNNYYLRLTAEMQDDIGTVGYVQFKNTDKKDFQKDDVFLALEASK 60
WP_046178069.1|GCSH_SDE              MKKFANYLLIEKKE--DRYTISMTPELQDDIGTIGYAEFTEND--TLAVDDIILNLEASK 56
                                     *.*    : :*     : * : :* : **:: ::   ::   :      :: :: :.::*

AEA00776.1|HMPREF9243_0576_AEUR      TVMEIQSPLKGSVVERHTEVVDQPSLLNSAKSEENWIVRLKDVDPAEFDALEEA------ 110
ADR24909.1|MBOVPG45_0459             EEKEFKMPIKASIIKINDSILAKPTLISSVYDDENWIATLKDINKEEYDKLDEFAMWDRY 120
WP_004420912.1|MBVG_4610             AILTLKMPFDAKVVEWNNAALAKPSLVSSHKDSENWIMIISDINPEILANLEDF------ 114
ACU79130.1|MMCAP1_0466               AILNLKMPLDATVVKWNQKALENPKLISSHDDNENWIMVLSDIDQNKFMSLEDF------ 114
AGJ91024.1|MPUT9231_6310             AILTLKMPLDATVVEWNQQIIENPELISSADQNNNWIMILTNIDKKTFDSLEDF------ 114
WP_046178069.1|GCSH_SDE              TVMSVLTPLAGTVVERNEAATLTPTLLNSEKADENWIVVLADVDQAAFDVLEDA------ 110
                                         .  *: ..::: .      * *:.*   .:***  : :::      *::

AEA00776.1|HMPREF9243_0576_AEUR      --------        110
ADR24909.1|MBOVPG45_0459             QSLISLIS        128
WP_004420912.1|MBVG_4610             --------        114
ACU79130.1|MMCAP1_0466               --------        114
AGJ91024.1|MPUT9231_6310             --------        114
WP_046178069.1|GCSH_SDE              --------        110
```

AEUR, *Aerococcus urinae;* MMCAP1, *M. mycoides* subsp.capri;, MPUT9231, *M. putrefaciens,* SDE, *Streptococcus dysgalactiae* subspecies *equisimilis*

Figure S1. Multiple sequence alignments of proteins (referred to in Table S2) that were assigned new enzyme annotations in this study for *M. bovis*. Multiple sequence alignments were constructed for the assigned proteins in *M. bovis* with homologous proteins identified in other mycoplasma and bacterial species using Clustal Omega at http://www.ebi.ac.uk/Tools/msa/clustalo/. Numbers on right indicate the position of the adjacent amino acid residue. An asterisk (*) indicates an amino acid that is conserved in all aligned sequences, a colon (:) indicates one substitution with a very similar amino acid, and a full stop (.) indicates at least one substitution with a similar amino acid. Dashed lines (-) indicate gaps in the amino acid sequence alignment. The active site for each enzyme is highlighted in red.
