# Supplementary material for: Comparative Metabolomics of Mycoplasma bovis and Mycoplasma gallisepticum Reveals Fundamental Differences in Active Metabolic Pathways and Suggests Novel Gene Annotations
Source: mSystems. 2017 Oct 10;2(5):e00055-17. doi: 10.1128/mSystems.00055-17 (PMC5634790; doi:10.1128/mSystems.00055-17)
Supplement: TABLE S2 [file sys005172140st2.pdf]

Table S2. Novel putative enzymes annotated onto the *M. bovis* metabolic map in this study.

Enzyme name	Enzyme abbr.	EC number	Gene number	Pathway	Equation	Evidence
A. Phosphotransferase glucose transporter subunitA<br>Phosphotransferase glucose transporter subunit B	PTSI/G	2.7.1.69	MBOVPG45_0417<br>MBOVPG45_0573	Glycolysis	Glucose + protein N(pi)-phospho L-histidine = Glucose 6-phosphate + protein histidine	1) Sequence homology (Fig. S1)
B. Phosphotriesterase	ULAG	3.1.1.-	MBOVPG45_0738	Ascorbate metabolism	L-ascorbate 6-phosphate + H_2O = 3-dehydro-L-gulonate 6-phosphate	1) Ortholog of *M. pneumoniae* phosphotriesterase (1, 2)<br>2) Genomic context<br>3) Gap filling
C. D-allulose 6-phosphate 3-epimerase	ALSE	5.1.3.-	MBOVPG45_0627	Fructose and mannose metabolism	D-Allulose 6-phosphate = beta-D-Fructose	1) Ortholog of *E.coli* alsE<br>2) Homology in crystal structure in *E. coli* alsE<br>3) Alternative activity for this enzyme in *E. coli* (3)
D. Carbohydrate uptake ABC transporter-2 family	CUT-2	-	MBOVPG45_0018<br>MBOVPG45_0019<br>MBOVPG45_0020	Carbohydrate uptake	A Carbohydrate + ATP + H_2O → A Carbohydrate + ADP + phosphate	1) Biocyc database search<br>2) Operon structure<br>3) Conserved domain search
E. Pseudouridine kinase	PSUK	2.7.1.83	MBOVPG45_0026	Pyrimidine metabolism	ATP + Pseudouridine = ADP + Pseudouridine 5'-phosphate	1) Ortholog of *E. coli psuK*<br>2) Conserved domain search
F. Fructose-bisphosphate aldolase	FBA	-	MBOVPG45_0088	Pentose phosphate pathway	Sedoheptulose-1,7-phosphate = glycerine phosphate + erythrose-4-phosphate	1) Enzymatic reaction annotated in *M. gallisepticum*(4)<br>2) Alternative activity of this enzyme in yeast (5)
G. Acetaldehyde dehydrogenase	ADH	1.2.1.10	MBOVPG45_0543	Pyruvate metabolism	Acetyl-CoA +NADH + H+ = acetaldehyde + CoA + NAD^+	1) Ortholog of *M. pnemoniae* MPN564 (2)<br>2) Alternative activity for this enzyme in *Thermoanaerobacter ethonolicus* (6)<br>3) Sequence homology (Fig. S1)
H. Glycerol ABC transporter	GTS A-2, B-2, C-2	-	MBOVPG45_0748<br>MBOVPG45_0749<br>MBOVPG45_0750	Glycerol uptake	Glycerol (out) + ATP = glycerol (in) + ADP	1) BioCyc database search

I. sn-glycerol-3-phosphate tranport system	UGP A, C, E	-	MBOVPG45_0309<br>MBOVPG45_0308<br>MBOVPG45_0307	sn-glycerol 3-phosphate uptake	sn-glycerol-3phosphate (out) + ATP = sn-glycerol 3-phosphate (in) + ADP	1) BioCyc database search<br>2) Orthologs with other mycoplasma species<br>2) Genomic context
J. Dihydroxyacetone kinase	DHAK	2.7.1.29	MBOVPG45_0523	Glycerolipid metabolism	Glycerone + ATP = Glycerone phosphate + ADP	1) Ortholog of *M. pneumoniae* dihydroacetone kinase<br>2) Conserved domain search<br>3) Sequence homology (Fig. S1)
K. Glycosyl transferase	GTF	2.4.1.157	MBOVPG45_0064	Glycerolipid metabolism	diacylglycerol + UDP-glucose = 3-D-glucosyl-1,2 diacyl-sn-glycerol + UDP	1) Ortholog of *M. pneumoniae* yibD (2)<br>2) Enzymatic activity (7)
L. Phosphatididylcholine acylhydrolase	PLDB	3.1.1.5	MBOVPG45_0331	Lipid metabolism	Phosphatidylcholine + 2 H_2O = sn-glycero 3-phosphocholine + 2 fatty acid	1) Ortholog of *M. pneumoniae* MPN445 (2)
M. Choline ethanolamine kinase	CHK	2.7.1.32	MBOVPG45_0333<br>MBVOPG45_0335	Glycerophospholipid metabolism	Choline + ATP = Choline-phosphate + ADP	1) Ortholog of *M. pneumoniae* MPN532 (2)<br>2) Conserved domain search
N. Choline-phosphate cytidylyltransferase	PCT	2.7.7.15	MBOVPG45_0084	Glycerophospholipid metabolism	Choline-phosphate + CTP = CDP-choline + Diphosphate	1) Ortholog of *M. pneumoniae* MPN336 (2)<br>2) Conserved domain search
O. Hypoxanthine phosphoribosyltransferase	HPT	2.4.2.8	MBOVPG45_0476	Purine metabolism	PRPP + Guanine = GMP + diphosphate	1) Enzymatic reaction in *M. pneumoniae* and *M. gallisepticum* (2, 4)
P. Adenine phosphoribosyltransferase	APT	2.4.2.7	MBOVPG45_0796	Purine metabolism	PRPP + Adenine = AMP + diphosphate	1) Enzymatic reaction in *M. pneumoniae* and *M. gallisepticum* (2, 4)
P. Orotate phosphoribosyltransferase	APT	2.4.2.10	MBOVPG45_0796	Pyrimidine metabolism	Orotidine 5'-phosphate + diphosphate = orotate + PRPP	1) Conserved domain analysis<br>2) Protein structure prediction
Q. 5' –nucleotidase	NT5 (ushA)	3.1.3.5	MBOVPG45_0690	Purine metabolism	AMP + H_2O = adenosine + phosphate<br>IMP + H_2O = Inosine+ phosphate<br>XMP + H_2O = Xanthosine + phosphate	1) Ortholog of *E. coli* ushA<br>2) Sequence homolog (Fig. S1)<br>3) Conserved domain search

					GMP + H_2O = Guanosine + phosphate	
					dGMP + H_2O = Deoxyguanosine + phosphate	
					dAMP + H_2O = Deoxyadenosine + phosphate	
Q. 5' –nucleotidase	NT5 (USHA)	3.1.3.5	MBOVPG45_0690	Pyrimidine metabolism	CMP + H_2O = Cytidine + Orthophosphate	1) Ortholog of *E. coli ushA*<br>2) Sequence homology (Fig. S1)<br>3) Conserved domain search
					UMP + H_2O = Uridine + Orthophosphate	
					dCMP + H_2O = Deoxycytidine + Orthophosphate	
					dUMP + H_2O = Deoxyuridine + Orthophosphate	
					dTMP + H_2O =Thymidine + Orthophosphate	
Q. Nicotinamide ribonucleotide phosphohydrolase	USHA	3.1.3.5	MBOVPG45_0690	Nicotinate and Nicotinamide metabolism	Nicotinate D-ribonucleotide + H_2O = Nicotinate D-ribonucleoside + Orthophosphate	1) Gap filling<br>2) Ortholog of *E. coli ushA*
					Nicotinamide D-ribonucleotide + H_2O = Nicotinamide-beta-riboside + Orthophosphate	
R. Pyruvate kinase	PYK	-	MBOVPG45_0158	Pyrimidine metabolism	UTP + pyruvate = UDP + phosphoenolpyruvate	1) Ortholog of *M. pneumoniae* pyrk MPN303<br>2) Enzymatic reaction (2, 4)
					CTP + pyruvate = CDP + phosphoenolpyruvate	
					dCTP + pyruvate = dCDP + phosphoenolpyruvate	
					dUTP + pyruvate = dUDP + phosphoenolpyruvate	

					dTTP + pyruvate = dTDP + phosphoenolpyruvate	
S. Orotidine 5'-phosphate decarboxylase	PYRF	4.1.1.23	MBOVPG45_0734	Pyrimidine metabolism	Orotidine 5'-phosphate + H^+ = CO2 + UMP	1) Gap filling<br>2) BioCyc database search
T. Pyrimidine phosphorylase<br>Uridine phsphorylase	PDP (UPP)	2.4.2.3	MBOVPG45_0301	Pyrimidine metabolism	Uridine + phosphate = Uracil + alpha -D-ribose 1-phoaphate	1) Gap filling<br>2) BioCyc database search<br>3) Ortholog of *M. pneumoniae* MPN064<br>4) Enzymatic activity (2, 8, 9)
		2.4.2.4			Thymidine +Phosphate = Thymine + 2-deoxy -alpha -D-ribose - 1 phosphate	
U. Malate transporter	AEC family	-	MBOVPG45_0327	Malate uptake	(R,S)-Malate (out) + H^+ = (R, S)-malate (in) + H^+	1) Ortholog of *M. fermentans* malate permease MFE_01530<br>2) Ortholog of *Clostridium cadaveris* malate permease protein<br>3) BioCyc and BLAST database search
V. Dicarboxylate/amino acid cation symporter family amino acid transporter	DAACS family	-	MBOVPG45_0568	Amino acid and dicarboxylate uptake	Substrate (dicarboxylate or amino acid) (out) + $nM^+(M^+=H^+$ or $Na^+)$ (out) = Substrate (In) + nM+(in)	1) Conserved domain search<br>2) Protein structure of a member of the DAACS family (10, 11)<br>3) Biocyc, Uniprot, transporter classification database search
W. Neutral amino acid transporter, L-type amino acid transporter	LAT family	-	MBOVPG45_0533	Amino acid uptake	Amino acid (out) = amino acid (in)	1) BLAST database search<br>2) Conserved domain search
X. Glycine cleavage system H protein	GCSH	-	MBOVPG45_0459	Amino acid metabolism	Glycine + H_4folate + NAD^+ = 5,10-methylene-H_4folate + CO_2 + NH_3 + NADH + H^+	1) Conserved domain search<br>2) Sequence homology (Fig. S1)
Y. Acyl carrier protein (ACP) phosphodiesterase	ACPH	3.1.4.14	MBOVPG45_0347	Pantothenate and CoA bioxynthesis	Acyl-carrier protein + H_2O = Pantetheine 4'-phosphate + Apo-[acyl-carrier-protein]	1) Ortholog of *M. pneumoniae* MPN479
Z. Pyridine nucleotide transhydrogenase, soluble	STHA	1.6.1.1	MBOVPG45_0108	Nicotinate and Nicotinamide metabolism	NADPH + NAD^+ = $NADP^+$ + NADH	1) Ortholog of *M. hyopneumoniae pdhD*-1 MHP168_678<br>2) Ortholog of *E. coli sthA* with *M. hyopneumoniae pdhD*-1<br>3) Conserved domain search
