# Supplementary material for: Comparative Metabolomics of Mycoplasma bovis and Mycoplasma gallisepticum Reveals Fundamental Differences in Active Metabolic Pathways and Suggests Novel Gene Annotations
Source: mSystems. 2017 Oct 10;2(5):e00055-17. doi: 10.1128/mSystems.00055-17 (PMC5634790; doi:10.1128/mSystems.00055-17)
Supplement: TABLE S3 [file sys005172140st3.pdf]

Table S3. Novel putative enzymes annotated onto the *M. gallisepticum* metabolic map in this study.

Enzyme name	Enzyme abbr.	EC number	Gene number	Pathway	Equation	Evidence
a. Phosphoglucomutase	PGM	5.4.2.2	MGA_0358	Glycolysis	alpha-D-glucose 1-phosphate = D-glucose 6-phosphatalphate	1) Ortholog of *M. pneumoniae* MPN066
	DeoB	5.4.2.7	MGA_0358	Pentose phosphate pathway	D-ribose-1-phosphate = D-ribose-5-phosphate	1) Ortholog of *M. pneumoniae* MPN066<br>2) Reaction was annotated in *M. pnuemoniae* MPN0622
b. Ribose ABC transporter	RibAB C	-	MGA_0365<br>MGA_0368<br>MGA_0372	Ribose uptake	Ribose (out) + ATP = Ribose (in) + ADP + phosphate	1) Ortholog of *M. pneumoniae* MPN258-260 (1)<br>2) Sequence homology (Fig. S2)<br>3) Conserved domain search
c. Dihydroxyacetone kinase	DHAK	-	MGA_0183		Glycerone + ATP = Glycerone phosphate + ADP	1) Ortholog of M. pneumonia MPN547<br>2) Sequence homology (Fig. S2)<br>3) Biocyc database search<br>4) Conserved domain search
d. Nucleoside disphosphate kinase	NDK	2.7.4.6	MGA_0156	Pyrimidine metabolism	ATP + nucleoside diphosphate = ADP + nucleoside triphosphate	1) Ortholog of *M. pneumoniae* MPN303
e. Uridine phosphorylase	UDP	2.4.2.3	MGA_0362	Pyrimidine metabolism	Uridine + phosphate = uracil + alpha-D-ribose 1-phosphate	1) Ortholog of *M. pneumoniae* MPN064<br>2) Conserved domain search<br>3) BioCyc database search<br>4) Enzymatic activity (1, 8, 12)
f. Malate dehydrogenase	MDH	1.1.1.37	MGA_0746	TCA cycle	(S)-malate + NAD^+ = oxaloacetate + NADH + H^+	1) Ortholog of *E.coli* MDH<br>2) Protein structure prediction Phyre2<br>3) BioCyc database search
g. Glycerol uptake facilitator protein	GlpF	-	MGA_0641	Glycerol uptake	Glycerol (out) = Glycerol (in)	1) Ortholog of *M. pneumoniae* GlpF MPN043
h. sn-glycerol-3-phosphate ABC transport system	UGP C, A, E	-	MGA_0677<br>MGA_0680<br>MGA_0682	sn-glycerol 3-phosphate uptake	Glycerol 3-phosphae (out)+ ATP = Glycerol 3-phosphate (in) + ADP + phosphate	1) Orthologs with MPN133-136<br>2) Genomic context

i. Glycosyl transferase	GTF	2.4.1.157	MGA_0343	Glycerolipid metabolism	Diacylglycerol + UDP-glucose = 3-D-glucosyl-1,2 diacyl-sn-glycerol + UDP	1) Ortholog of *M. pneumoniae* yibD MPN483<br>2) Enzymatic activity (1, 7)
j. Phosphatidylcholine acylhydrolase	PLDB	3.1.1.5	MGA_0999	Lipid metabolism	Phosphatidylcholine + 2 H2O = sn-glycero 3 phosphocholine + 2 fatty acid	1) Ortholog of *M. pneumoniae* MPN445
k. Choline ethanolamine kinase	CHK	2.7.1.32	MGA_0931	Glycerophospho lipid metabolism	Choline + ATP = Choline-phosphate + ADP	1) Ortholog of *M. pneumoniae* MPN532<br>2) Conserved domain search
l. Choline-phosphate cytidylyltransferase	PCT	2.7.7.15	MGA_0052	Glycerophospho lipid metabolism	Choline-phosphate + CTP = CDP-choline + Diphosphate	1) Ortholog of *M. pneumoniae* MPN336<br>2) Conserved domain search
m. Amino acid permease	-	-	MGA_0287	Amino acid uptake	Amino acid (out) = amino acid (in)	1) BioCyc database search<br>2) Conserved domain search
n. NAD^+ kinase	NADK	2.7.1.23	MGA_0291	Nicotinate and Nicotinamide metabolism	NADH +ATP = NADPH + ADP	1) Ortholog of MPN267<br>2) Enzymatic activity (1,9)
o. Riboflavine kinase	RFK	2.7.1.26	MGA_0832	Riboflavin metabolism	ATP + FMN + H^+ = FAD + diphosphate	1) BioCyc database search<br>2) Conserved domain search
					Riboflavin + ATP = ADP + FMN + H+	
p. Pantetheine phosphate adenylyltransferase	COASY	2.7.7.3	MGA_0052	Panthothenate and CoA biosynthesis	ATP + pantetheine 4'-phosphate = diphosphate + 3'-dephospho-CoA	1) Ortholog of *M. pneumoniae* MPN336<br>2) Conserved domain search
q. Acyl carrier protein (ACP) phosphodiesterase	ACPH	3.1.4.14	MGA_0243	Pantothenate and CoA bioxynthesis	Acyl-carrier protein + H_2O = Pantetheine 4'-phosphate + Apo-[acyl-carrier-protein]	1) Ortholog of *M. pneumoniae* MPN479<br>2) Conserved domain search

TEXT 1. Reference list for Tables S2 and S3.

1. **Boudker, O., R. M. Ryan, D. Yernool, K. Shimamoto, and E. Gouaux.** 2007. Coupling substrate and ion binding to extracellular gate of a sodium-dependent aspartate transporter. Nature **445:**387-93.

2. **Burdette, D., and J. G. Zeikus.** 1994. Purification of acetaldehyde dehydrogenase and alcohol dehydrogenases from Thermoanaerobacter ethanolicus 39E and characterization of the secondary-alcohol dehydrogenase (2° Adh) as a bifunctional alcohol dehydrogenase-acetyl-CoA reductive thioesterase. Biochem J **302 (Pt 1):**163-70.

3. **Chan, K. K., A. A. Fedorov, E. V. Fedorov, S. C. Almo, and J. A. Gerlt.** 2008. Structural basis for substrate specificity in phosphate binding (β/α)8-barrels: D-allulose 6-phosphate 3-epimerase from *Escherichia coli* K-12. Biochemistry **47:**9608-9617.

4. **Horecker, B. L., P. Z. Smyrniotis, H. H. Hiatt, and P. A. Marks.** 1955. Tetrose phosphate and the formation of sedoheptulose diphosphate. J Biol Chem **212:**827-36.

5. **Klement, M. L., L. Ojemyr, K. E. Tagscherer, G. Widmalm, and A. Wieslander.** 2007. A processive lipid glycosyltransferase in the small human pathogen *Mycoplasma pneumoniae*: involvement in host immune response. Mol Microbiol **65:**1444-57.

6. **Maier, T., J. Marcos, J. A. Wodke, B. Paetzold, M. Liebeke, R. Gutierrez-Gallego, and L. Serrano.** 2013. Large-scale metabolome analysis and quantitative integration with genomics and proteomics data in *Mycoplasma pneumoniae*. Mol Biosyst **9:**1743-55.

7. **McElwain, M. C., and J. D. Pollack.** 1987. Synthesis of deoxyribomononucleotides in Mollicutes: dependence on deoxyribose-1-phosphate and PPi. J Bacteriol **169:**3647-53.

8. **Mcgarrity, G. J., L. Gamon, T. Steiner, J. Tully, and H. Kotani.** 1985. Uridine Phosphorylase-Activity among the Class Mollicutes. Current Microbiology **12:**107-112.

9. **Mori, S., S. Kawai, F. Shi, B. Mikami, and K. Murata.** 2005. Molecular conversion of NAD kinase to NADH kinase through single amino acid residue substitution. J Biol Chem **280:**24104-12.

10. **Vanyushkina, A. A., G. Y. Fisunov, A. Y. Gorbachev, D. E. Kamashev, and V. M. Govorun.** 2014. Metabolomic analysis of three Mollicute species. PLoS One **9:**e89312.

11. **Yernool, D., O. Boudker, Y. Jin, and E. Gouaux.** 2004. Structure of a glutamate transporter homologue from *Pyrococcus horikoshii*. Nature **431:**811-8.

12. **Yus, E., T. Maier, K. Michalodimitrakis, V. van Noort, T. Yamada, W. H. Chen, J. A. Wodke, M. Guell, S. Martinez, R. Bourgeois, S. Kuhner, E. Raineri, I. Letunic, O. V. Kalinina, M. Rode, R. Herrmann, R. Gutierrez-Gallego, R. B. Russell, A. C. Gavin, P. Bork, and L. Serrano.** 2009. Impact of genome reduction on bacterial metabolism and its regulation. Science **326:**1263-8.
