# Supplementary material for: Comparative Metabolomics of Mycoplasma bovis and Mycoplasma gallisepticum Reveals Fundamental Differences in Active Metabolic Pathways and Suggests Novel Gene Annotations
Source: mSystems. 2017 Oct 10;2(5):e00055-17. doi: 10.1128/mSystems.00055-17 (PMC5634790; doi:10.1128/mSystems.00055-17)
Supplement: FIG S2 [file sys005172140sf8.pdf]

1) Ribose ABC transporter

M. pneumoniae used as reference. No known active site.

```
NP_109948.1|MPN260                  -----------------------------------------------------MSQSLIS 7
AAP56937.2|MGA_0365                 ---------------------------------------------------------MFS 3
EGZ31650.1|GUU_00867                ---------------------------------------------------------MFD 3
WP_040094460.1|SMSRO_RS09145        MQKQIKSYGWLLRQKTKIFFRSNEFKTKMNYFKASVCAIIAGAILSFIIIGANASNPLLF 60
                                                                                         ::

NP_109948.1|MPN260                  F---------SNLD-NWLFVAPALLLAVLSGYLSERVGIVNIAINGGMVFGGMFLSLMSY 57
AAP56937.2|MGA_0365                 P---------TQLD-QWLILAPALILGVFGGYLSERVGIVNIAINGTMTFGATFFVLFSN 53
EGZ31650.1|GUU_00867                F---------AQLFDIALLFFAVLSLGSLSGFFSERAGIVNIGINGMMIFGALFYTIFGG 54
WP_040094460.1|SMSRO_RS09145        FSYVFRLAFHPLLKDQTLTYWAIYIVAGLAVAVGFKAGLFNIGVPGQMLLAGSMTIVLGL 120
                                                *    *       :. :.  .. :.*:.**.: * * :.. :  ::.

NP_109948.1|MPN260                  AFVPNA-----NDSAPSWSLAISIPLSVIFASAVGFLFGIAAIKLKADHVIVGTG----- 107
AAP56937.2|MGA_0365                 VFFRSMG--GMLTTTYNWTFLASILLSALLSVPVGILFGVATIKLKADHVIAGTG----- 106
EGZ31650.1|GUU_00867                LMNGGSLGNNLGGNAGNWTFFIAMILASLCTIVVGLLFGYSTIKLKADHIIAGTA----- 109
WP_040094460.1|SMSRO_RS09145        KNPAISQGAGVV-----GALLISILVGAALAAIAGALKAYFNIHEVVSTIMLNWIVWYVM 175
                                                      ::  :: :.   :  .* * .   *:  .. ::

NP_109948.1|MPN260                  -VNLLGTGINFFVAQNA-RSLLNDTDLRVRYSFVRTGNSV--SIEGIAIFAFAIIFVLLV 163
AAP56937.2|MGA_0365                 -INLLATGIGQILSDRA-TTLFGRPSLDNVYNRTLRPNSV--STEAIIIFVLFIVFFIIL 162
EGZ31650.1|GUU_00867                -INIFGAAIGMFLTNSLGKTLTGNDQLTNPYKEFWKIGDGFFFGSSLLIFFVVLALIVAL 168
WP_040094460.1|SMSRO_RS09145        KWMFMNPAHGMWNSNHNS-TI---DIVTAAPNFNLTLN----GQTWIIPFIIAMLLLLTA 227
                                       ::  .     ::    ::     :    .              :  * . : :.:

NP_109948.1|MPN260                  WYLMNFTKTGLRYRAVGENPNVIDTQGISVYKYQWFGVMASTMVAALAGCCFALSP-QVP 222
AAP56937.2|MGA_0365                 YVVMNFSKIGLRYRAVGENPNAVDSQGINVTKYQWIGIILASVVAGSGGALFGYYQ-SGR 221
EGZ31650.1|GUU_00867                YVVIKFTKFGLRFVSIGENPNAADSQGINVVKYQWIGIIIASMLAGLGGAIFMFTS-T-- 225
WP_040094460.1|SMSRO_RS09145        IFIMNYTVLGFRIKAVGKSRNASLYAGTNVRAYMIVSMALSGGLAGVLGMLYYMTQSTVL 287
                                      :::::  *:*  ::*:. *.    * .*  *  ..:  :  :*.  *  :

NP_109948.1|MPN260                  SFSSGDVSGFGFIAIAIMIISMWRIIPSIVISPLFALAYVLTTG-V------VGNANNTY 275
AAP56937.2|MGA_0365                 SF-AGDVDGVGFIALAILIVSGWKLLPITIIGLVFSSLLVHSTSTT------SVNQSQIF 274
EGZ31650.1|GUU_00867                SF-NGDVNGIGFLALAIMITGSWRIPLITIVSILFSLLVGFSKI--------GIQNVNSD 276
WP_040094460.1|SMSRO_RS09145        QFTTDALPVVGFDAIAVALVAFTNGVAILPIALLWGIIKTAALQATQLPDFQMSKQMGQL 347
                                    .*    :  .** *:*: : .  .      :. ::.     :            :

NP_109948.1|MPN260                  LLRTIPFIISLLVMMVFGYLNV---GPKNVGKHFDKGLR--------------------- 311
AAP56937.2|MGA_0365                 LVRTIPYALTLATMLLLGKFSI---GPKNVGNHFDKGLR--------------------- 310
EGZ31650.1|GUU_00867                VLKIIPYAASLVALVLFSKKIR---GPKFAGQHFDKSRR--------------------- 312
WP_040094460.1|SMSRO_RS09145        IFGIIIYMTAISALFIYFKPIFWISRWWNIRHHVDWKQEYDEYKQQIKTYQRQIKTINKR 407
                                    :.  * :  :: .:.:               .*.*   .

NP_109948.1|MPN260                  ------------------------------------------------------------ 311
AAP56937.2|MGA_0365                 ------------------------------------------------------------ 310
EGZ31650.1|GUU_00867                ------------------------------------------------------------ 312
WP_040094460.1|SMSRO_RS09145        YRVKIHELKQQGDKELIKAYREEINDELTLYAGKITTLKTEMRFFTNSKYKEAAKIGQRG 467

NP_109948.1|MPN260                  ------------------------------------------------------------ 311
AAP56937.2|MGA_0365                 ------------------------------------------------------------ 310
EGZ31650.1|GUU_00867                ------------------------------------------------------------ 312
WP_040094460.1|SMSRO_RS09145        IETKYDLAVFIALGSAMDHFVQVKNEYLLKKQQASFLKRNYVQTVKKCKGQTKQKLSQLY 527

NP_109948.1|MPN260                  ------------------------------------------------------------ 311
AAP56937.2|MGA_0365                 ------------------------------------------------------------ 310
EGZ31650.1|GUU_00867                ------------------------------------------------------------ 312
WP_040094460.1|SMSRO_RS09145        GLPKKDLYARLVQLQTQYGDLILKQEDAIKKLRESYYPVFDEIQQKYLTDFKTLQSKEKV 587

NP_109948.1|MPN260                  ------------------------------------------------------------ 311
AAP56937.2|MGA_0365                 ------------------------------------------------------------ 310
EGZ31650.1|GUU_00867                ------------------------------------------------------------ 312
WP_040094460.1|SMSRO_RS09145        VTKQLNQEIKKLKHQQWKEMCQFKITNYQTRKEIKHELKAIDVKLKNDPGIQKEVMMLKK 647

NP_109948.1|MPN260                  ------------------------------------------------------------ 311
AAP56937.2|MGA_0365                 ------------------------------------------------------------ 310
EGZ31650.1|GUU_00867                ------------------------------------------------------------ 312
WP_040094460.1|SMSRO_RS09145        DLTIQLTTMKQQFDVENKVVKEKHRTNLPQWRNELNQKLNTIIKAVGEDNKILKTEYLRQ 707

NP_109948.1|MPN260                  --------------    311
AAP56937.2|MGA_0365                 --------------    310
EGZ31650.1|GUU_00867                --------------    312
WP_040094460.1|SMSRO_RS09145        KSVYHKSAQEGGKN    721
```

MPN, *M. pneumoniae*; MGA, *M. gallisepticum*; GUU, *M. iowae*, SMSRO, *Spiroplasma poulsonii*

2) Dihydroxyacetone kinase (DHAK)

Streptococcus salivarius used as reference. No known active site.

```
WP_002887028.1|SSAL                 --MSKITTSLLQEMVQAAATRLGKQAEYVNSLNVFPVPDGDTGTNMGMTMDNGAKAVAD-        57
AAP56845.2|MGA_0183                 MASDTLTTKQLQSMFIEGYNVIFNRYEYINNLNVFPVPDGDTGTNMKITTKGGVDAISD-        59
WP_010874904.1|MPN547               --MSTVNLSNFIDMLRLGCKNIANNFEYINQLNVFPVPDGDTGTNMKVTLSEAFKKLESE        58
WP_011077239.1|MYPE4130             --MKSLETKILVSMFENGAKEIAKHYEYINELNVFPVPDGDTGTNMKITATSAVEALNKQ        58
WP_004025236.1|GUU_03959            --MKTIDSKKFLDMFGAGCQQITDSCDYINELNVFPVPDGDTGTNLKITTNSAYESTLQK        58
```

```
                                       ..:  . : .*.  .   : .  :*:*.**************: :*   . .   .

WP_002887028.1|SSAL          -QSASTVGEVGQILSKGLLMGARGNSGVITSQLFRGFGQSIKD-----KTELDGQDLAHA        111
AAP56845.2|MGA_0183          -LEFNSLQSLGKVFATGLFMNACGNSGVIFSQIIKGFTKSLPD----GD-EVGIKQLINS        113
WP_010874904.1|MPN547        ISHIKSFSDLGKSFTRDLLLFSRGNSGVIFSQIMKGFFSDMISTKTATETELGIEDFATA        118
WP_011077239.1|MYPE4130      LPELGSIYEVGKFFCRQLLMNARGNSGVIFSQIIRGFFEPIEE----STKKLTIELIVRC        114
WP_004025236.1|GUU_03959     ISDNSTFVEITSILSRQLLMNARGNSGVIFSQIFRGFFDSIKE----ENNKLDCDLLKKC        114
                                  :. .: . :   *:: : ****** **:::** . : .       ::  . :  .

WP_002887028.1|SSAL          FQTGVEVAYKAVMKPVEGTILTVSRGAASAAIKKAESTNDAVEVMRAALDGAKAALAKTP        171
AAP56845.2|MGA_0183          FKEAKEVAYSVIQSPVEGTILTVVRLTADQLVQKQDEISSVEQLFELATEFAKDALKQTP        173
WP_010874904.1|MPN547        FIKAEEVAYKNVSKPVEGTMLTVIRLISTDFKNQKNRAKTVQKLFEQVIKTAWQTVKKTP        178
WP_011077239.1|MYPE4130      FISAKEKAYKSISNPVEGTILTIIREISEQLPK--ENINDVEELFKKALTIGLNALEKTP        172
WP_004025236.1|GUU_03959     LISAKERAYRSITNPIEGTILTIIRVISEEMQT--KEIDDIEHFFKEIVSVGFDILKKTP        172
                             :  . * **  : .*:***:**: *  :        .  .   ..:.     .   : :**

WP_002887028.1|SSAL          EMLPVLKEVGVVDSGGQGLVFIYEGFLSALTGEYIASEDFQATPATM----TEMIN---A        224
AAP56845.2|MGA_0183          KMLPALKSANVVDSGGFGLVSFLEGMLTELTKDFSRLNND--------KQF-------VN        218
WP_010874904.1|MPN547        QMLPVLKASGVVDSGAYGFACFLEGMLSFYGEKATLNDGK----LTS-----------AE        223
WP_011077239.1|MYPE4130      EILPSLKEAKVVDSGGYGLCKFLEGMYNQLVNSDDDNVQNKDIEIEKKKETVESISVTSK        232
WP_004025236.1|GUU_03959     EMLISLKEAKVVDSGGYGLCKFFEGMYLCVSGSLVTDKSN--EKLKKDESDIKDITINSK        230
                             ::*   **   *****. *:  : **:       .

WP_002887028.1|SSAL          EHHKSVAGHVATEDITFGYCTEIMIGLK----QGPTYVKDFDYEEFRNYLSNLGDSLLVV        280
AAP56845.2|MGA_0183          KKALSSKAEQELVEEGHGYCTEFLLKLGLKAKDD-QTKIKFNEEKFKKEISKDVESLVLI        277
WP_010874904.1|MPN547        LSQMTISGEKHVTEEEFGYCTEYVLKLGMSVSQE-VEKQKFNQKKFESKVSKIATSVVVA        282
WP_011077239.1|MYPE4130      NTFIQSKVRNDISEEGFGYCCEFIIQKDFITHEKQPLKIKFDLNKFEKEMLLAGESLVVV        292
WP_004025236.1|GUU_03959     NTFINNQSRSEISEEGFGYCCEFIIQQNFITYDGQKEKEKFNKNSFEKELLAFGDSLIVV        290
                                     .   :  .*** * ::       :      .*: :.*.. :     *:::

WP_002887028.1|SSAL          ND--DEIVKVHVHTEDPGLVMQEGLKYGALVKVKVDNMRNQHDAQVQKEEAIQAAPSAPK        338
AAP56845.2|MGA_0183          NDKEEGIVKLHAHTLTPDLVLKHAQPYGEFLKVKIENMNKQVTERKQDDSNLGQLANKPI        337
WP_010874904.1|MPN547        SDKDNGFVKVHAHTEKPNLLLELGLNYGEFELVKIENMNLQVAKQKPAPVK---------        333
WP_011077239.1|MYPE4130      DD--SDIVKVHIHSITPSLVFEIGQKYGEFLKLKVENMTLQYIESHPGVS--------PS        342
WP_004025236.1|GUU_03959     TD--EDIIKVHMHALEPYKVLLVGQKYGEFLKIKVENMTQQYLDTHPEIN--------PK        340
                              *  . ::*:* *:  *  ::  .  ** :  :*::**  *

WP_002887028.1|SSAL          DF----------------ALIAVVAGDGLADIF-KSQGVDYVISGGQTMNPSTEDIVKAI        381
AAP56845.2|MGA_0183          DLLMDIDLSKIKDFKTETAIIATVPSRVLGKMIVQNYDVDQFIDTSATGNPTIKDFVKNI        397
WP_010874904.1|MPN547        -------------RNIKPAIVVTVPTEAFADRIREDYDIQAILCTDDTGAPSVFSLLEAV        380
WP_011077239.1|MYPE4130      DL------FNKFSLTDDVQIVATCPSRKIADYLKNEFGINNVIVTEETGNPSTNEIMNQI        396
WP_004025236.1|GUU_03959     DM------FKTTQLVDDVKLVVTLPSQKISEYYLEELNIENSIITEDTGNPSTNDILNSI        394
                                                  :::..     :..   :.  ::  :    *  *:  .::: :

WP_002887028.1|SSAL          EQVNAKNVIILPNNKNIFMAAQSAAEVVD---VNAAVVETRTVPQGFTSLLAFDPSQSIE        438
AAP56845.2|MGA_0183          YSVKSKNVIIVVDDTNLLLSAKEAIALLKK-HINCELISCQNFIEALSAISGYMRSDSLK        456
WP_010874904.1|MPN547        KLTHSSNIIFLLHDKNYFLSANEALKQLKHQKISADCVMTTNPIESLAALTVFNSDLNIH        440
WP_011077239.1|MYPE4130      INCKSRNILVLTDDSNIILSAEQAATFMKE-RYDVVVLKSKNIIEVINACLVFSSEKSIS        455
WP_004025236.1|GUU_03959     KDTKSKNVIVVTDDSNIILSAEQTVELLKN-KVNVSIVRGKNLMESMLACLNFNSSLSIY        453
                                .: *::.: .:.* :::*:.:   :.     .   :    .  : : :   :  . .:

WP_002887028.1|SSAL          ANVEAMTASLSDVTSGSVTLAVRDTTIDGLEIHENDILGMVDGKILVSTPDMDQALLDTF        498
AAP56845.2|MGA_0183          SNVKVLHKIIKSTGSAFISTSVKNIKYPHLQVHKGDYIGVMKKKIIVSDSDIYKCLLDTV        516
WP_010874904.1|MPN547        TNVKTMRRFVKGFASATITQASKKYKENRIEVNKGDFIAVANNSICVSEKELVQCVFNTI        500
WP_011077239.1|MYPE4130      ENARVMNRAIRKQDSAMLSVSVKTSKFNGVHVEKNDFIGIYDKKIVLAEKNSEIAITNLI        515
WP_004025236.1|GUU_03959     ENQKEMNKVVKKSITGLISKSVKTAKFNDVMVQKGDNIGILDKRIVLSEKSTESAAISLI        513
                              * . :   :     :. :: : :  .    : :.: * :.: .   * ::  .    .  . .

WP_002887028.1|SSAL          DKMIDEDS--EIVMIYVGEEGNQEQAQALAEKLEEAHEGIEVEIFQGDQPVYPYLFSVE 555
AAP56845.2|MGA_0183          AQLIDEVKKPELVLIIYGKNTSSSEVRTIV-KLISEKFGIYCEAINGSQKLYQYYIGVQ 574
WP_010874904.1|MPN547        DHLLKKVKKPEFLLAYYGKDITAEEAEAMK-EKIEKKYKLFCEFSPGEQKVFSYILGIQ 558
WP_011077239.1|MYPE4130      KKLRDRNKKAQLLYIVYGQGVTLREIRNIE-KFANENYGIKCKLIDGGQKIYRYFIGMS 573
WP_004025236.1|GUU_03959     KKMKAKIRKPEMVYLIYGQGISLREVRNIE-KFVNENYGIKIKAVSGNQKLYQYYIGIM 571
                              ::  .     :::    *:  .  : . :  :  . .  :  :    * * :: * :.:
```

SSAL, *Streptococcus salivarius*; MYPE4130, *M. penetrans*; GUU, *M. iowae*

Figure S2. Multiple sequence alignments of proteins (referred to in Table S3) that were assigned new enzyme annotations in this study for *M. gallisepticum*. Multiple sequence alignments were constructed for the assigned proteins in *M. gallisepticum* with homologous proteins identified in other mycoplasma and bacterial species using Clustal Omega at http://www.ebi.ac.uk/Tools/msa/clustalo/. Numbers on right indicate the position of the adjacent amino acid residue. An asterisk (*) indicates an amino acid that is conserved in all aligned sequences, a colon (:) indicates one substitution with a very similar amino acid, and a full stop (.) indicates at least one substitution with a similar amino acid. Dashed lines (-) indicate gaps in the amino acid sequence alignment.
