# Supplementary material for: Comparative Metabolomics of Mycoplasma bovis and Mycoplasma gallisepticum Reveals Fundamental Differences in Active Metabolic Pathways and Suggests Novel Gene Annotations
Source: mSystems. 2017 Oct 10;2(5):e00055-17. doi: 10.1128/mSystems.00055-17 (PMC5634790; doi:10.1128/mSystems.00055-17)
Supplement: FIG S3 [file sys005172140sf9.pdf]

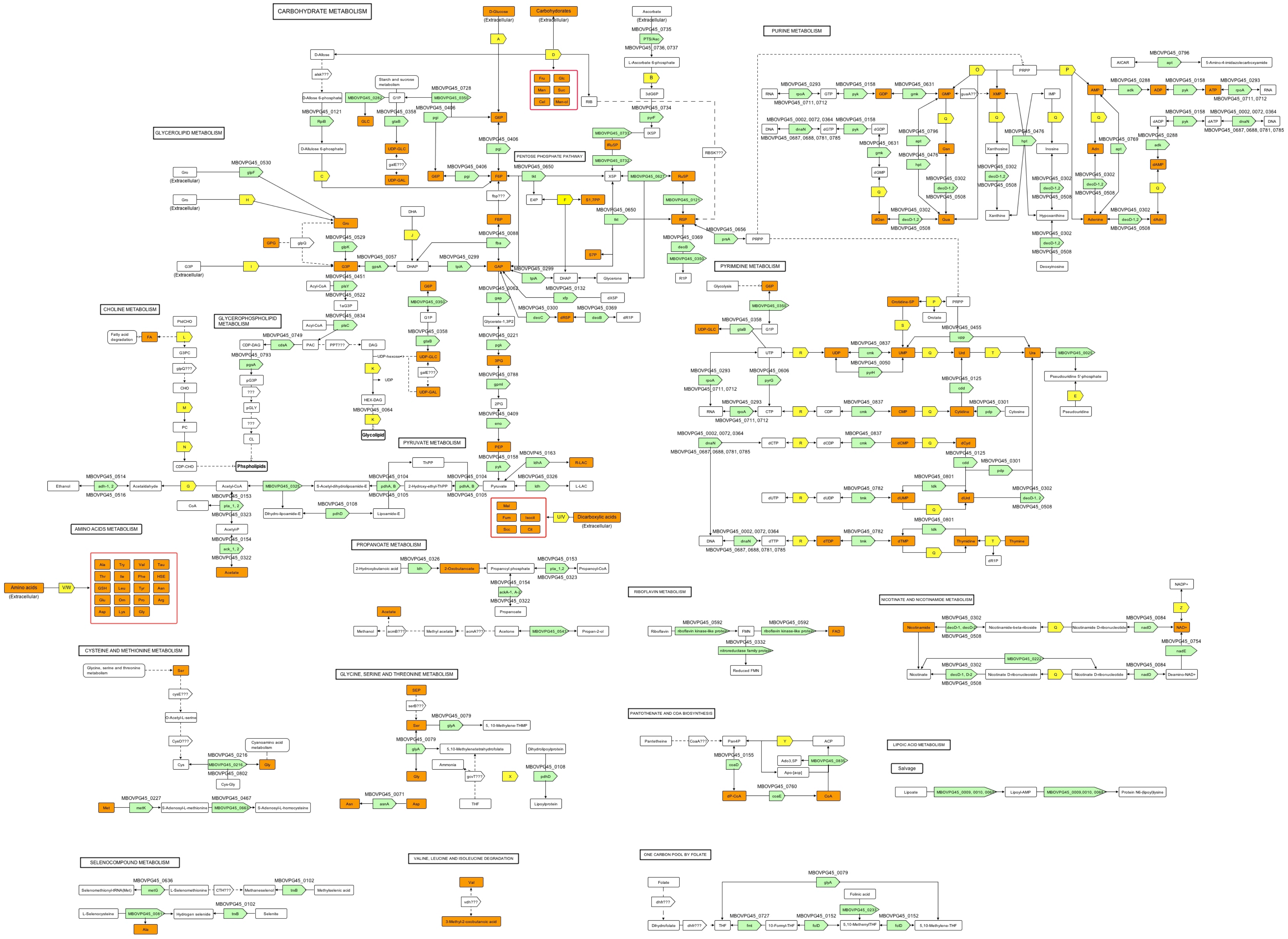

CARBOHYDRATE METABOLISM
D-Glucose
(Extracellular)
Carbohydorates
(Extracellular)
Ascorbate
(Extracellular)
MBOVPG45_0735
PTS/Asc
MBOVPG45_0736, 0737
L-Ascorbate 6-phosphate
3dG6P
MBOVPG45_0734
pyrF
MBOVPG45_0733
IX5P
IRu5P
MBOVPG45_0732
D-Allose
alsk???
D-Allose 6-phosphate
MBOVPG45_0121
RpiB
D-Allulose 6-phosphate
Fru
Glc
Man
Suc
Cel
Man-ol
RIB
RBSK???
Starch and sucrose metabolism
G1P
MBOVPG45_0282
MBOVPG45_0350
MBOVPG45_0728
GLC
MBOVPG45_0358
gtaB
UDP-GLC
galE???
UDP-GAL
MBOVPG45_0406
pgi
G6P
F6P
fbp???
FBP
MBOVPG45_0088
fba
PENTOSE PHOSPHATE PATHWAY
MBOVPG45_0650
tkt
X5P
MBOVPG45_0627
Ru5P
E4P
S1,7PP
MBOVPG45_0121
R5P
S7P
GLYCEROLIPID METABOLISM
Gro
(Extracellular)
MBOVPG45_0530
glpF
GPG
glpQ
MBOVPG45_0529
glpK
G3P
MBOVPG45_0057
gpsA
DHAP
DHA
MBOVPG45_0299
tpiA
GAP
MBOVPG45_0451
plsY
Acyl-CoA
MBOVPG45_0522
1aG3P
MBOVPG45_0834
plsC
PAC
PPT???
DAG
UDP-hexose
UDP
HEX-DAG
MBOVPG45_0064
Glycolipid
CHOLINE METABOLISM
PtdCHO
Fatty acid degradation
FA
G3PC
glpQ???
CHO
PC
CDP-CHO
Phspholipids
GLYCEROPHOSPHOLIPID METABOLISM
MBOVPG45_0749
CDP-DAG
cdsA
MBOVPG45_0793
pgsA
pG3P
pGLY
CL
MBOVPG45_0062
gap
Glycerate-1,3P2
MBOVPG45_0221
pgk
3PG
MBOVPG45_0788
gpmI
2PG
MBOVPG45_0409
eno
PEP
MBOVPG45_0158
pyk
MBOVPG45_0132
xfp
dX5P
Glycerone
MBOVPG45_0300
deoC
dR5P
MBOVPG45_0369
deoB
dR1P
R1P
MBOVPG45_0656
prsA
PRPP
PYRUVATE METABOLISM
ThPP
MBOVPG45_0163
ldhA
R-LAC
MBOVPG45_0326
ldh
L-LAC
Pyruvate
MBOVPG45_0104
pdhA, B
MBOVPG45_0105
2-Hydroxy-ethyl-ThPP
S-Acetyl-dihydrolipoamide-E
MBOVPG45_0325
Acetyl-CoA
Acetaldehyde
adh-1, 2
MBOVPG45_0514
MBOVPG45_0516
Ethanol
MBOVPG45_0153
pta_1, 2
CoA
MBOVPG45_0323
Acetyl-P
MBOVPG45_0154
ack_1, 2
MBOVPG45_0322
Acetate
Dihydro-lipoamide-E
MBOVPG45_0108
pdhD
Lipoamide-E
Mal
Fum
Isocit
Scc
Cit
U/V
Dicarboxylic acids
(Extracellular)
AMINO ACIDS METABOLISM
Amino acids
(Extracellular)
V/W
Ala
Try
Val
Tau
Thr
Ile
Phe
HSE
GSH
Leu
Tyr
Asn
Glu
Orn
Pro
Arg
Asp
Lys
Gly
PROPANOATE METABOLISM
2-Hydroxybutanoic acid
2-Oxobutanoate
Propanoyl phosphate
Propanoyl-CoA
ackA-1, A-2
Propanoate
Methanol
acmB???
Methyl acetate
acmA???
Acetone
MBOVPG45_0543
Propan-2-ol
CYSTEINE AND METHIONINE METABOLISM
Glycine, serine and threonine metabolism
Ser
cysE???
O-Acetyl-L-serine
CysO???
Cys
Cyanoamino acid metabolism
MBOVPG45_0216
MBOVPG45_0802
Gly
Cys-Gly
MBOVPG45_0227
Met
metK
S-Adenosyl-L-methionine
MBOVPG45_0467
MBOVPG45_0663
S-Adenosyl-L-homocysteine
GLYCINE, SERINE AND THREONINE METABOLISM
SEP
serB???
MBOVPG45_0079
glyA
5, 10-Methylene-THMP
5,10-Methylenetetrahydrofolate
Ammonia
gcvT???
THF
Dihydrolipoylprotein
pdhD
Lipoylprotein
MBOVPG45_0071
asnA
Asn
SELENOCOMPOUND METABOLISM
MBOVPG45_0636
Selenomethionyl-tRNA(Met)
metG
L-Selenomethionine
CTH???
Methaneselenol
MBOVPG45_0102
trxB
Methylselenic acid
L-Selenocysteine
MBOVPG45_0081
Hydrogen selenide
Selenite
VALINE, LEUCINE AND ISOLEUCINE DEGRADATION
vdh???
3-Methyl-2-oxobutanoic acid
PURINE METABOLISM
MBOVPG45_0796
AICAR
apt
5-Amino-4-imidazolecarboxamide
RNA
rpoA
GTP
GDP
gmk
GMP
guaA???
XMP
IMP
AMP
adk
ADP
ATP
MBOVPG45_0293
MBOVPG45_0711, 0712
MBOVPG45_0631
MBOVPG45_0288
MBOVPG45_0002, 0072, 0364
MBOVPG45_0687, 0688, 0781, 0785
DNA
dnaN
dGTP
dGDP
dGMP
dGsn
Gsn
Gua
hpt
MBOVPG45_0476
MBOVPG45_0302
MBOVPG45_0508
deoD-1,2
Xanthosine
Xanthine
Inosine
Hypoxanthine
Deoxyinosine
Adn
Adenine
dAdn
dAMP
dADP
dATP
MBOVPG45_0769
PYRIMIDINE METABOLISM
Glycolysis
MBOVPG45_0358
UDP-GLC
UTP
UDP
UMP
MBOVPG45_0837
cmk
MBOVPG45_0050
pyrH
Urd
Ura
MBOVPG45_0025
Orotidine-5P
Orotate
MBOVPG45_0455
upp
Pseudouridine 5'-phosphate
Pseudouridine
MBOVPG45_0606
pyrG
CTP
CDP
CMP
MBOVPG45_0125
cdd
Cytidine
MBOVPG45_0301
pdp
Cytosine
dCTP
dCDP
dCMP
dCyd
MBOVPG45_0801
tdk
dUTP
dUDP
dUMP
dUrd
MBOVPG45_0782
tmk
dTTP
dTDP
dTMP
Thymidine
Thymine
RIBOFLAVIN METABOLISM
Riboflavin
MBOVPG45_0592
riboflavin kinase-like protein
FMN
FAD
MBOVPG45_0332
nitroreductase family protein
Reduced FMN
NICOTINATE AND NICOTINAMIDE METABOLISM
Nicotinamide
deoD-1, deoD-2
Nicotinamide-beta-riboside
Nicotinamide D-ribonucleotide
MBOVPG45_0084
nadD
NAD+
NADP+
MBOVPG45_0754
nadE
MBOVPG45_0222
Nicotinate
Nicotinate D-ribonucleoside
Nicotinate D-ribonucleotide
Deamino-NAD+
PANTOTHENATE AND COA BIOSYNTHESIS
Pantetheine
CoaA???
Pan4P
ACP
MBOVPG45_0155
coaD
Ado3,5P
MBOVPG45_0835
Apo-[acp]
MBOVPG45_0760
dP-CoA
coaE
CoA
LIPOIC ACID METABOLISM
Salvage
Lipoate
MBOVPG45_0009, 0010, 0068
Lipoyl-AMP
Protein N6-(lipoyl)lysine
ONE CARBON POOL BY FOLATE
Folate
dhfr???
Dihydrofolate
THF
MBOVPG45_0727
fmt
10-Formyl-THF
MBOVPG45_0152
folD
Folinic acid
MBOVPG45_0233
5,10-MethenylTHF
5,10-Methylene-THF

Figure S3. Complete metabolic map of *M. bovis* constructed in this study. Orange boxes indicate detected metabolites. Yellow boxes indicate novel annotations from this study. Green boxes indicate previous annotations in the KEGG database (39).
