# Supplementary material for: Comparative Metabolomics of Mycoplasma bovis and Mycoplasma gallisepticum Reveals Fundamental Differences in Active Metabolic Pathways and Suggests Novel Gene Annotations
Source: mSystems. 2017 Oct 10;2(5):e00055-17. doi: 10.1128/mSystems.00055-17 (PMC5634790; doi:10.1128/mSystems.00055-17)
Supplement: FIG S4 [file sys005172140sf10.pdf]

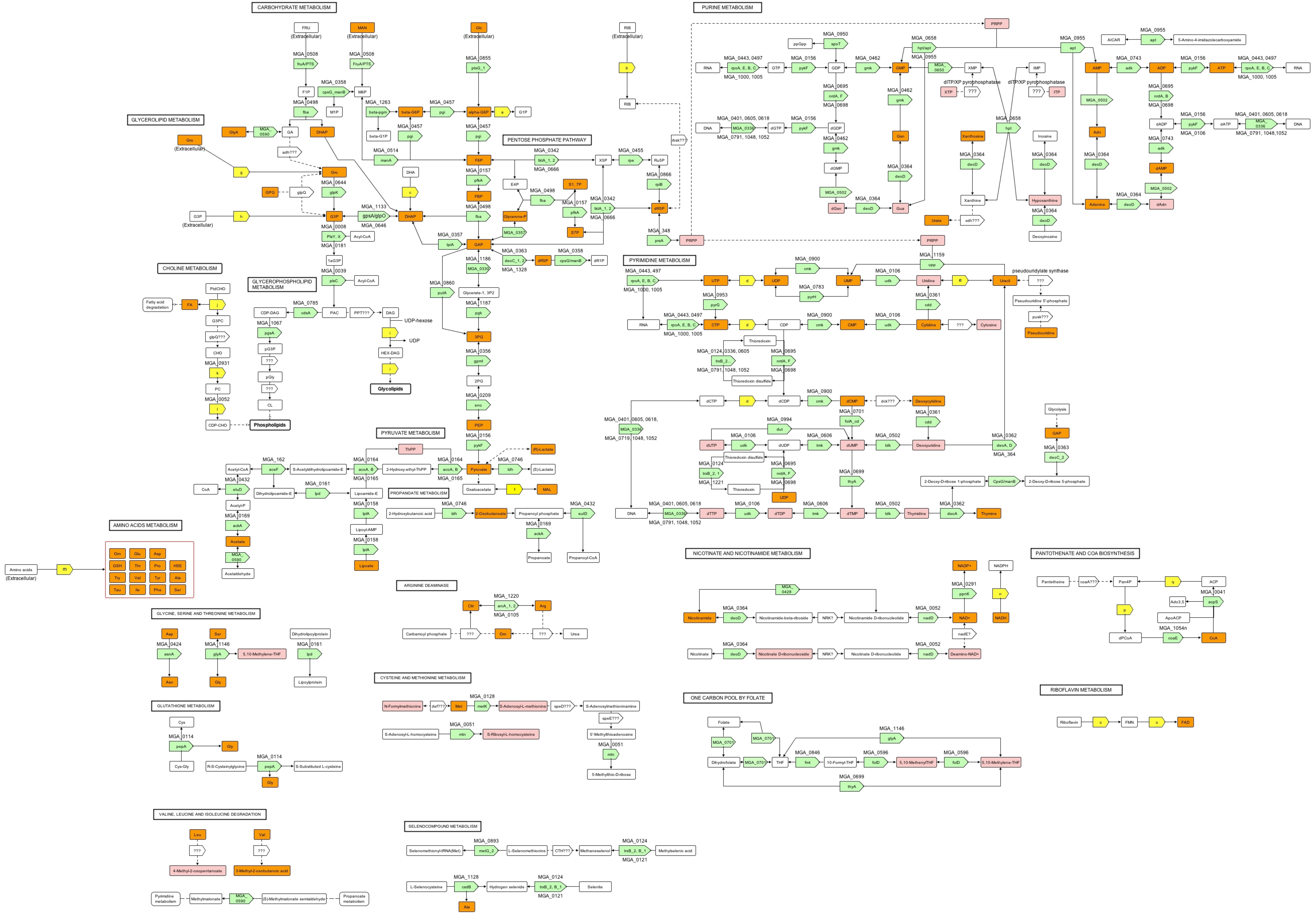
CARBOHYDRATE METABOLISM
PURINE METABOLISM
GLYCEROLIPID METABOLISM
PENTOSE PHOSPHATE PATHWAY
CHOLINE METABOLISM
GLYCEROPHOSPHOLIPID METABOLISM
PYRIMIDINE METABOLISM
PYRUVATE METABOLISM
PROPANOATE METABOLISM
AMINO ACIDS METABOLISM
ARGININE DEAMINASE
NICOTINATE AND NICOTINAMIDE METABOLISM
PANTOTHENATE AND COA BIOSYNTHESIS
GLYCINE, SERINE AND THREONINE METABOLISM
CYSTEINE AND METHIONINE METABOLISM
GLUTATHIONE METABOLISM
ONE CARBON POOL BY FOLATE
RIBOFLAVIN METABOLISM
VALINE, LEUCINE AND ISOLEUCINE DEGRADATION
SELENOCOMPOUND METABOLISM

Figure S4. Complete metabolic map of *M. gallisepticum* constructed in this study (updated from map inferred by Vanyushkina et al (4)). Orange boxes indicate detected metabolites. Pink boxes indicate previously detected metabolites (4). Yellow boxes indicate novel annotations from this study. Green boxes indicate previous annotations in the KEGG database (39).
