# Supplementary material for: Comparative Metabolomics of Mycoplasma bovis and Mycoplasma gallisepticum Reveals Fundamental Differences in Active Metabolic Pathways and Suggests Novel Gene Annotations
Source: mSystems. 2017 Oct 10;2(5):e00055-17. doi: 10.1128/mSystems.00055-17 (PMC5634790; doi:10.1128/mSystems.00055-17)
Supplement: TABLE S4 [file sys005172140st4.pdf]

Table S4. Metabolite abbreviations used in Figures 1, 2 and 3.

Abbreviation	Metabolites
Glc	D-Glucose
G1P	D-Glucose 1-phosphate
G6P	D-Glucose 6-phosphate
F6P	Fructose 6-phosphate
FBP	Fructose 1,6-bisphosphate
GAP	DL-Glyceraldehyde 3-phosphate
3PG	3-Phosphoglycerate
PEP	Phophoenolpyruvate
Fru	D-Fructose
Man-ol	D-Mannitol
Man	D-Mannose
Suc	Sucrose
Cel	Cellobiose
Rib	Ribose
GlcN	D-Glucosamine
GlcA	Glucuronic acid
Man-6-P	D-Mannopyranose 6-phosphate
Gal-6-P	D-Galactofuranose 6-phosphate
Sor-6-P	D-Sorbitol 6-phophate
GlcN1P	D-Glucosamine 1-phosphate
Allo-Ino	Allo-inositol
Glyc	Glyceric acid
DHA	glycerone, dihydroxyacetone
DHAP	glycerone phosphate, dihydroxyacetone phosphate
3dG6P	3-dehydro-L-gulonate 6-phosphate

E4P	D-Erythrose 4-phosphate
R5P	D-Ribose 5-phosphate
Ru5P	D-Ribulose 5-phosphate
X5P	D-Xylulose 5-phosphate
lX5P	L-Xylulose 5-phosphate
lRu5P	L-Ribulose 5-phosphate
dR5P	2-Deoxy-D-ribose 5-phosphate
dX5P	D-Xylulose 5-phosphate
dR1P	2-Deoxy-D-ribose 1-phosphate
S1,7P	Sedoheptulose 1,7-phosphate
S7P	Sedoheptulose 7-phosphate
dR5P	2-Deoxy-D-ribose 5-phosphate
2dhglcn	2-Dehydro-D-Gluconate
GalL	D-Galactono-1,4-lactone
PRPP	5-phospho-D-ribose 1-diphosphate
Citr	Citrate
Isocit	Isocitrate
Fum	Fumarate
Mal	Malate
Lac	Lactate
OAA	Oxaloacetate
Orn	Ornithine
Citr	Citrulline
Lys	Lysine
Trp	L-Tryptophan
Gly	Glycine
Pro	L-Proline
Asn	L-Asparagine

Arg	Arginine
Asp	Aspartic.acid
Ala	Alanine
Tyr	L-Tyrosine
Glu	L-Glutamic acid
Thr	L-Threonine
GSH	Glutathione
Phe	Phenylalanine
Leu	Leucine
Ile	Isoleucine
Tau	Taurine
Met	Methionine
Cys	Cysteine
Ser	Serine
SEP	O-phospho-L-serine
PCA	Pyroglutamate
GPG	103.101.28.709.01 alpha glycerphosphorylgrycerol
Gro	Glycerol
G3P	Glycerol 3-phosphate
2obut	2,Oxobutanoate
1aG3P	1-Acyl-sn-glycerol 3-phosphate
PAC	Phosphatidate, 1,2-diacyl-sn-glycerol 3-phosphate
DAG	Diacylglycerol, 1,2-diacyl-sn-glycerol
CDP-DAG	CDP-diacylglycerol
HEX-DAG	HEX-diacylglycerol
pG3P	Phosphatidylglycerophosphate, 3(3-sn-phosphatidyl)-sn-glycerol 1-phosphate
pGLY	Phosphatidylglycerophosphate, 3(3-sn-phosphatidyl)-sn-glycerol 1-phosphate
CL	Cardiolipin

PtdCHO	Phosphatidylcholine
G3PC	sn-Glycero-3-phosphocholine
CHO	Choline
PC	Choline phosphate
CDP-CHO	CDP-Choline
Ttdca	Tetradecanoate (Myristic acid)
Hdca	Hexadecanoate (Palmitic acid)
Ade	Adenine
Adn	Adenosine
AMP	Adenosine 5'-monophosphate
ADP	Adenosine 5'-diphosphate
ATP	Adenosine 5'-triphosphate
dAMP	Deoxyadenosine 5'-monophosphate
dADP	Deoxyadenosine 5'-diphosphate
dATP	Deoxyadenosine 5'-triphosphate
IMP	Inosine 5'-monophosphate
Xan	Xanthine
Xtsn	Xanthosine
XMP	Xanthosine 5'-monophosphate
Gsn	Guanosine
GMP	Guanosine 5'-monophosphate
GDP	Guanosine 5'-diphosphate
GTP	Guanosine 5'-triphosphate
dGMP	Deoxyguanosine 5'-monophosphate
dGDP	Deoxyguanosine 5'-diphosphate
dGTP	Deoxyguanosine 5'-triphosphate
CMP	Cytidine 5'-monophosphate
CDP	Cytidine 5'-diphosphate

CTP	Cytidine 5'-triphosphate
Dcyd	Deoxycytidine
dCMP	Deoxycytidine 5'-monophosphate
dCDP	Deoxycytidine 5'diphosphate
dCTP	Deoxycytidine 5'-triphosphate
dTMP	Deoxythymidine 5'-monophosphate
dTDP	Deoxythymidine 5'-diphosphate
dTTP	Deoxythymidine 5'-triphosphate
Urd	Uridine
Ura	Uracil
UMP	Uridine 5'-monophosphate
UDP	Uridine 5'-diphosphate
UTP	Uridine 5'-triphosphate
dUMP	Deoxyuridine 5'-monophosphate
dUDP	Deoxyuridine 5'-diphosphate
dUTP	Deoxyuridine 5'-triphosphate
Uac	Uric acid
Psu	Pseudouridine
UDP-Glc	UDP-Glucose
UDP-Gal	UDP-Galactose
P	Phosphoric acid
FMN	Flavin mononucleotide
FAD	Flavin-adenine dinucleotide
NAD	Nicotinamide adenine dinucleotide
NADH	Nicotinamide adenine dinucleotide -reduced
NADP	Nicotinamide adenine dinucleotide phosphate
4PA	4-Pyridoxic acid
Pan4P	Pantetheine 4-phosphate

ACP	acyl-carrier-protein, holo[acyl-carrier-protein]
Ado3,5P	Adenosine 3',5'-bisphosphate
dPo-CoA	Dephosphocoenzyme A
apo(acp)	apo-[acyl-carrier protein]
THF	Tetrahydrofolate
