# Supplementary material for: Comparative Metabolomics of Mycoplasma bovis and Mycoplasma gallisepticum Reveals Fundamental Differences in Active Metabolic Pathways and Suggests Novel Gene Annotations
Source: mSystems. 2017 Oct 10;2(5):e00055-17. doi: 10.1128/mSystems.00055-17 (PMC5634790; doi:10.1128/mSystems.00055-17)
Supplement: TABLE S5 [file sys005172140st5.pdf]

**Table S5**. Level of metabolite identification for metabolites detected on GC/MS

| Metabolite | Retention time (min) | Fragmented ion | Level of identification |
|---|---|---|---|
| AMP | 14.933 | 169 | 1 |
| Adenine | 10.894 | 264 | 1 |
| Sucrose | 13.445 | 361 | 1 |
| D-Glucose | 10.861/10.956 | 319 | 1 |
| Talose/D-Mannose | 10.809 | 319 | 1 |
| Fructose | 10.743 | 307 | 1 |
| Trehalose | 13.78 | 361 | 1 |
| Myoinositol | 11.661 | 305 | 1 |
| Glucose-6P | 12.336 | 357 | 1 |
| Glycerol-3P | 10.281 | 243 | 1 |
| 3-Phospho-D-glycerate | 10484 | 357 | 1 |
| Frucose-6P | 12.38 | 315 | 1 |
| Ribulose-5P | 11.63/11.69 | 340/221 | 1 |
| Glyceric acid | 8.173 | 189 | 1 |
| D-Lactic acid | 6.757 | 117 | 1 |
| Fumaric acid | 8.317 | 245 | 1 |
| Malic acid | 9 | 233 | 1 |
| Isocitrate | 10.545 | 245 | 1 |
| Acetic acid | 9.032 | 232 | 1 |
| L-Ornithine | 10.57 | 142 | 1 |
| L-Lysine | 11.003 | 317 | 1 |
| Tryptophan | 12.228 | 202 | 1 |
| L-Valine (IS) | 7.59 | 144 | 1 |
| Aspartic acid | 8.731/9.179 | 232 | 1 |
| Phenylalanine | 9.455/9.776 | 120 | 1 |
| L-Histidine | 11.046 | 254 | 1 |
| L-Serine | 8.337 | 204 | 1 |
| L-Proline | 8.083 | 142 | 1 |
| Glycine | 7.189/8.109 | 102/248 | 1 |
| L-Alanine | 6.991 | 116 | 1 |
| Isoluecine | 8.016 | 158 | 1 |
| L-Asparagine | 9.91 | 231 | 1 |
| L-Threonine | 8.042/8.479 | 117 | 1 |
| L-Homoserine | 8.82 | 128 | 1 |
| L-Methionine | 9.228 | 176 | 1 |
| Tyrosine | 11.101 | 280 | 1 |
| Myristic acid | 10.73 | 285 | 1 |
| Palmitic acid | 11.592 | 313 | 1 |
| Stearic acid | 12.257 | 341 | 1 |
