# Supplementary material for: Comparative Metabolomics of Mycoplasma bovis and Mycoplasma gallisepticum Reveals Fundamental Differences in Active Metabolic Pathways and Suggests Novel Gene Annotations
Source: mSystems. 2017 Oct 10;2(5):e00055-17. doi: 10.1128/mSystems.00055-17 (PMC5634790; doi:10.1128/mSystems.00055-17)
Supplement: TABLE S6 [file sys005172140st6.pdf]

Table S6. Level of metabolite identification for metabolites detected on LC/MS with authenticated standards

Metabolite	Retention time	Accurate mass	Obtained mass	ppm error	Level of identification
Cholesteryl sulfate	1.109	465.3034	465.30439	2.13	2
Myristic acid	1.225	227.2015	227.2015133	0.06	1
4-Pyridoxic acid	1.457	182.0458	182.0748878	159.78	2
Uracil	2.769	111.0198	111.0198336	0.30	1
Adenine	3.647	134.0466	134.0471054	3.77	1
Biotin	4.4	243.0801	243.0628296	-71.05	2
Deoxycytidine	5.039	226.0827	226.0830372	1.49	1
Phenylalanine	5.136	164.0713	164.0711262	-1.06	1
Pyroglutamic acid	5.17	128.0354	128.0354362	0.28	1
D-Galactono-1,4-lactone	5.401	177.0401	177.0408104	4.01	1
D-Glucosamine	5.57	178.0715	178.0373009	-192.05	2
Leucine	5.965	130.0872	130.0871399	-0.46	1
Cytidine	6.084	242.0774	242.0893372	49.31	2
L-Tryptophan	6.214	203.082	203.0784241	-17.61	2
Uridine	6.28	243.0614	243.0605659	-3.43	1
Tryptophan	6.5	203.0824	203.0784241	-19.58	1
Betaine	6.68	116.0716	116.9164906	7279.05	2
L-Valine	6.695	116.0717	116.9164906	7278.18	2
Guanosine	6.72	282.0844	282.0780182	-22.62	2
Proline	6.796	114.0555	114.0558809	3.34	1
Uric Acid	6.927	167.0208	167.0390625	109.34	2
Tyrosine	7.3	180.0664	180.0664001	0.00	1
2-Oxobutanoate	7.374	101.0242	101.0415018	171.26	2
Mannitol	7.492	181.0709	181.1877888	645.54	2
Lactate	7.6	89.0245			2

D-fructose	7.953	179.0557	179.0557507	0.28	1
Glycine	8.03	74.0254			2
Alanine	8.182	88.0409			2
Histidine	8.238	154.0619	154.0619934	0.61	1
sn-Glycerol-3P	8.371	171.0059	171.0406616	203.28	2
dUMP	8.388	307.0331	307.0340622	3.13	1
AMP	8.503	346.0558	346.0559738	0.50	1
Glutathione	8.588	306.0758	306.0595459	-53.10	2
Asparagine	8.609	131.0463	131.0460869	-1.63	2
Glutamic acid	8.653	146.0456	146.0419224	-25.18	2
NADH	8.742	662.1009	662.1015106	0.92	1
Aspartic acid	8.808	132.0299	132.0303326	3.28	1
2-Deoxy-D-ribose-5P	8.808	213.0165	213.0167023	0.95	1
Cellobiose	8.818	341.1069	341.1089493	6.01	1
D-Galacturonate	9	193.035	193.0279968	-36.28	2
Citrulline	9.048	174.0875	174.1066437	109.97	2
Ornithine	9.067	131.0824	131.0824055	0.04	1
dCMP	9.101	306.0483	306.048793	1.61	1
UMP	9.166	323.0285	323.0286774	0.55	1
DL-Glyceraldehyde-3P	9.233	168.9909	168.990797	-0.61	1
GMP	9.3	362.0513	362.0517848	1.34	1
CMP	9.3	322.0436	322.0446846	3.37	1
2-Dehydro-D-gluconate	9.313	193.0344	193.0279968	-33.17	2
ADP	9.382	426.021	426.0226723	3.93	1
Fumarate	9.415	115.0033	115.0089157	48.83	2
Malate (Malic acid)	9.465	133.0138	133.0142193	3.15	1
D-Ribose-5P	9.5	229.0113	229.0123837	4.73	1
Malate	9.512	133.0138	133.0142193	3.15	1

UDP-D-Glucose	9.852	565.0471	565.0475337	0.77	1
O-phospho-L-serine	9.946	184.0011	184.0011215	0.12	1
D-Glucosamine-1P	9.968	258.0388	258.0381012	-2.71	1
ATP	9.97	505.9888	505.9889832	0.36	1
D-Glucose-6P	10.1	259.022	259.0222031	0.78	1
IDP	10.151	427.007	427.0075508	1.29	1
Phosphoenolpyruvate	10.526	166.9749	166.973777	-6.73	1
Isoleucine	10.758	191.0191			2
Isocitrate	10.758	191.0191			2
Citrate	10.772	191.0197			2
CTP	11.013	481.9758	481.126783	-1761.53	2
Fructose 1/6-bisphosphate	11.046	338.9877	338.994133	18.98	2
Lysine	12.862	145.0984	145.0982189	-1.25	1
Arginine	13.177	173.1042	173.1042419	0.24	1

A metabolite with ppm error<±10 is considered a level 1 identified metabolites.

A metabolite with ppm error>±10 is characterised as a level 2 putative compound.
